# Steroidogenic compensation and lipid deficiency with enhanced NAD ^+^ salvage in small‐for‐gestational‐age placenta

**DOI:** 10.1111/febs.70475

**Published:** 2026-03-10

**Authors:** Serena Xodo, Lorenza Driul, Vanessa Tolotto, Eros Di Giorgio, Luigi E. Xodo

**Affiliations:** ^1^ Clinic of Obstetrics and Gynecology Santa Maria della Misericordia Hospital, ASUFC 33100 Udine Italy; ^2^ Department of Medicine, Laboratory of Biochemistry University of Udine 33100 Udine Italy

**Keywords:** ω‐3/ω‐6 PUFAs, cholesterol uptake, NAD^+^ salvage pathway, SGA placenta, steroidogenesis

## Abstract

Fetal growth restriction (FGR) affects approximately 8% of pregnancies in Western countries and is characterised by complex placental adaptations at both metabolic and transcriptional levels. In this study, we integrated RNA sequencing and metabolomic analyses to investigate alterations in steroidogenesis, NAD^+^ metabolism and ω‐3/ω‐6 polyunsaturated fatty acid (PUFA) pathways in placental biopsies and trophoblast organoids. Placentas from small‐for‐gestational‐age (SGA10 and SGA3) infants, compared with appropriate‐for‐gestational‐age (AGA) controls, showed increased cholesterol uptake and enhanced steroid biosynthesis. In SGA3 placentas, these changes were accompanied by activation of the NAD^+^ salvage pathway, supporting elevated steroidogenesis, redox balance and energy metabolism. Despite this compensatory response, concentrations of key steroid metabolites, including androstenedione sulfate and oestrogens, were reduced. Metabolomic profiling further revealed a marked depletion of lysophospholipids enriched in ω‐3 and ω‐6 PUFAs, along with decreased levels of free arachidonic acid (ARA), docosahexaenoic acid (DHA) and selected prostaglandins and thromboxanes. These alterations suggest mobilisation of lipid stores to counteract reduced PUFA‐derived eicosanoid production, a process that may compromise placental vascular regulation and fetal neurodevelopment. Collectively, our results highlight the metabolic plasticity of the FGR placenta and identify coordinated alterations in lipid and NAD^+^ metabolism as key adaptive responses to placental insufficiency.

## Graphical abstract

Fetal growth restriction is associated with placental metabolic adaptations. In small‐for‐gestational‐age placenta (SGA), cholesterol receptors and steroidogenic enzymes are upregulated, enhancing steroidogenesis. NAD salvage pathway is also increased to support NADP^+^/NADPH requirements. Despite these adaptations, several steroid hormones are reduced in SGA placenta. Furthermore, metabolomic profiling revealed depletion of ω‐3 and ω‐6 PUFA‐enriched lysophospholipids, reduced free arachidonic (ARA) and docosahexaenoic (DHA) acids and lower levels of selected eicosanoids.

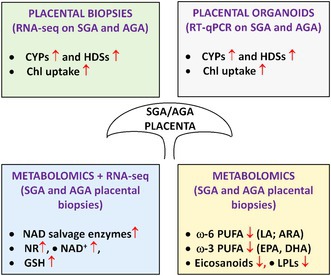

**Abbreviations**
AGA: appropriate‐ for‐ gestational age
ARA: arachidonic acid
Chl: cholesterol
DAF‐2DA: 4,5‐diaminofluorescein‐2‐diacetate
DAMs: differentially accumulated metabolites
DEGs: differentially expressed genes
DHA: docosahexaenoic acid
DHEA‐S: dehydroepiandrosterone sulphate
DMEM: Dulbecco modified Eagle medium
EVT: extravillous trophoblasts
FA: fatty acids
FGR: fetal growth restriction
GO: gene ontology
GSEA: gene set enrichment analysis
HSD: hydroxysteroid dehydrogenases
LC/MS: liquid chromatography/Mass spectrometry
LP: lysophospholipids
NA: nicotinic acid
NAM: nicotinamide
NES: normalised enrichment score
NMN: nicotinamide mononucleotide
NO: nitric oxide
NR: nicotinamide riboside
PlOs: placenta organoids
PPP: pentose phosphate pathway
PUFA: polyunsaturated fatty acid
RNA‐seq: RNA sequencing
RT‐qPCR: quantitative reverse transcription polymerase chain reaction
SGA10: small‐ for‐ gestational‐ age, birth weight <10th percentile
SGA3: small‐ for‐ gestational age, birth weight <3rd percentile
STBs: syncytiotrophoblasts
TOM: trophoblast organoid medium

## Introduction

The placenta plays a central role in fetal development by regulating the transport of nutrients from mother to fetus [1]. It is also characterised by intense bioenergetic and biosynthetic activity to support the rapid growth of the fetus. Lipids are important molecules for placental function, serving as a primary source of ATP, structural components of cell membranes and precursors for bioactive molecules such as prostaglandins, thromboxanes and leukotrienes [2, 3, 4, 5]. Moreover, in the second and third trimesters of pregnancy, the synthesis of steroid hormones is dominated by the placenta and the fetal adrenal glands [6]. The latter produce dehydroepiandrosterone sulphate (DHEA‐S) and its hydroxylated derivative, which are converted into oestrogens in the placenta [7, 8]. Placental steroidogenesis leading to the synthesis of progesterone depends on the availability of cholesterol (Chl), which is supplied to the placenta by the maternal and fetal circulation rather than being synthesised *de novo*. Disorders of lipid metabolism, such as altered fatty acid composition or enzyme activity, have been associated with pregnancy complications, including fetal growth restriction and pre‐eclampsia [7]. Polyunsaturated fatty acids (PUFAs), such as arachidonic acid (ARA, a 20:4 fatty acid) and docosahexaenoic acid (DHA, 22:6), are particularly important for the placenta as they contribute to cellular signalling pathways and fetoplacental growth. ARA acts as a precursor of eicosanoids [9, 10, 11].

Lipids and steroids are critical for normal fetal development and growth, and alterations in their profiles and impaired steroidogenic enzyme activity have been associated with placental dysfunction and adverse fetal outcomes [11, 12, 13]. A deeper understanding of lipid metabolism and steroidogenesis in the placenta is essential for identifying mechanisms underlying pregnancy complications and for developing possible targeted therapeutic interventions.

In this study, we employed an integrated transcriptomic and metabolomic approach to compare steroidogenesis and lipid metabolism in placentas from infants classified as appropriate‐for‐gestational‐age (AGA) and those classified as small‐for‐gestational‐age (SGA) [14]. SGA infants, defined as having birth weights below the 10th (SGA10) or 3rd percentile (SGA3) for gestational age, frequently experience fetal growth restriction, particularly in the case of SGA3, which is strongly associated with placental dysfunction [15]. While metabolomics reveals metabolic alterations linked to placental insufficiency, transcriptomics offers insight into the underlying molecular mechanisms by profiling genome‐wide gene expression. We aimed to determine whether lipid and steroid profiles differ between AGA and SGA placentas, with the goal of identifying molecular signatures specific to the SGA condition. These findings may enhance our understanding of the pathogenesis of FGR.

We found that the dysfunctional SGA3 placenta is characterised by altered steroidogenesis and disrupted levels of PUFAs and eicosanoids. Notably, the SGA3 placenta exhibits upregulation of genes encoding key steroidogenic enzymes, including CYP11A1, CYP19A1, HSD3B1, HSD17B1, and STS, as well as cholesterol transporters LDLR and SR‐BI. In addition, we observed significant activation of the NAD^+^ salvage pathway, along with marked downregulation of 20β‐dehydroprogesterone and estrone metabolites. The SGA3 placenta also showed deficiencies in essential PUFAs, such as arachidonic acid (AA, ω‐6), docosahexaenoic acid (DHA, ω‐3) and lysophospholipids (LPs) containing ω‐3 and ω‐6 fatty acids. Comparing steroidogenesis and lipid metabolism between AGA and SGA3 placentas is therefore essential to understanding the complex interplay between placental hormone production and fetal growth. Our findings offer valuable insights into the molecular mechanisms underlying fetal growth restriction and may be useful for designing future strategies to improve maternal and fetal health outcomes.

## Results

### RNA‐seq and metabolomic data analyses

We analysed 23 term placentas from both male and female newborns, categorised into two groups: appropriate‐for‐gestational‐age (AGA, *n* = 9) placentas and small‐for‐gestational‐age (SGA, *n* = 14) placentas. The SGA group was further subdivided into placentas from newborns with birth weight below the 10th percentile (SGA10, *n* = 9) and those with birth weight below the 3rd percentile (SGA3, *n* = 5) [14]. Detailed maternal characteristics are provided in Table S1.

Total RNA extracted from each placental biopsy was subjected to RNA sequencing (RNA‐seq) to generate transcriptome profiles for the AGA, SGA10 and SGA3 groups. Comparative analysis between AGA and SGA3 placentas identified 249 differentially expressed genes (DEGs), of which 182 were downregulated and 67 were upregulated (|log_2_ fold change| ≥ 1, *P* < 0.05) [16].

Gene Ontology (GO) enrichment analysis of the downregulated DEGs (FDR <0.05) in SGA3 placentas revealed an overrepresentation of processes associated with steroid and hormone metabolism, including steroid metabolic process (*P* < 10^−5^), steroid hormone biosynthesis (*P* < 10^−2^) and androgen biosynthesis (*P* < 10^−3^). Conversely, the upregulated DEGs were significantly enriched in terms associated with pre‐eclampsia and placental stress adaptation, such as pre‐eclampsia (PE) (*P* < 10^−6^), syncytiotrophoblast function (*P* < 10^−9^), hormone activity (*P* < 10^−6^), HIF‐1 signalling under hypoxia (*P* < 10^−3^) and hypoxia (*P* < 10^−9^). These results suggest that SGA3 placenta has a dual molecular signature: dysregulation of normal steroid and hormone metabolism, and activation of hypoxia‐ and stress‐related signalling pathways.

**Fig. 1:**
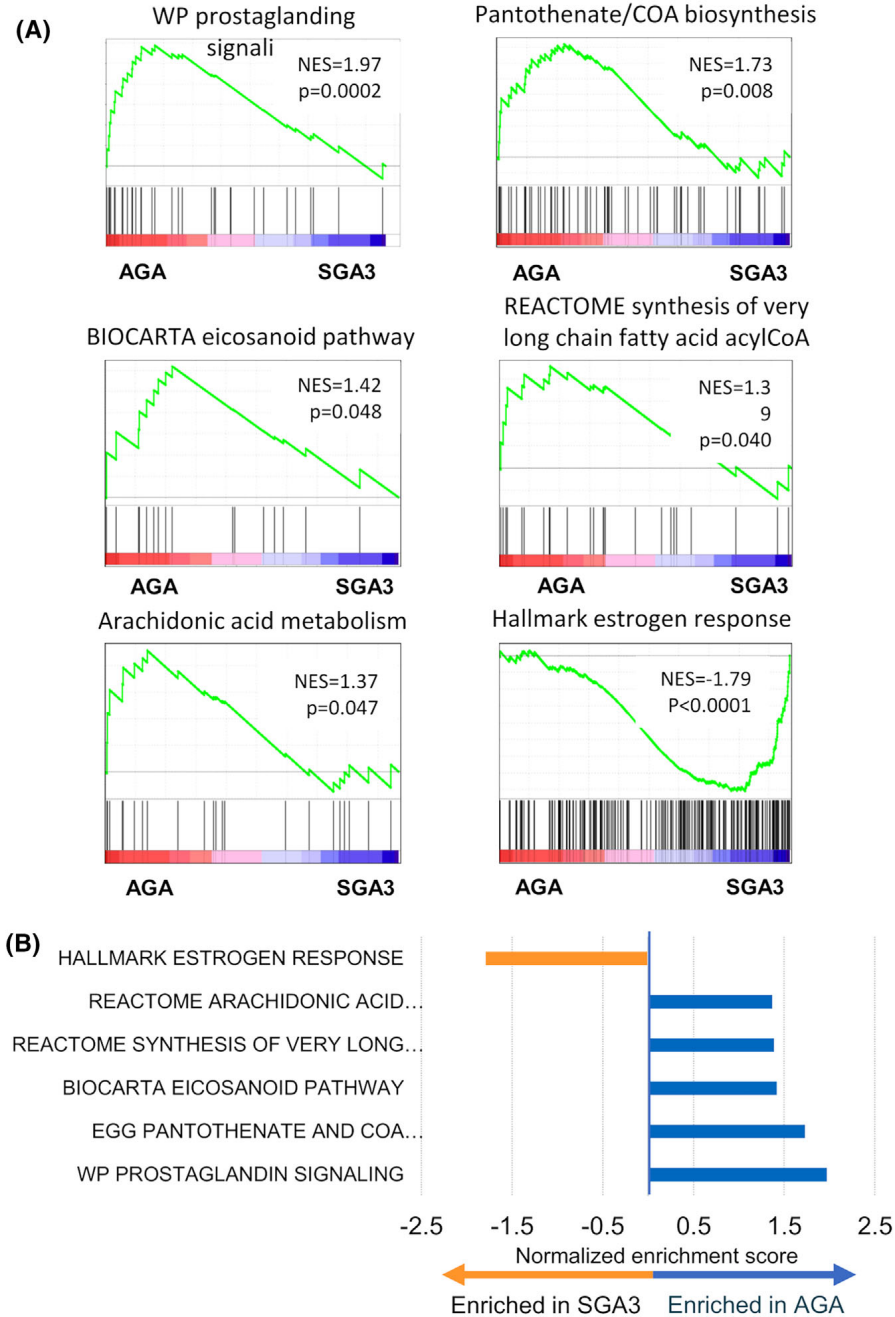
Gene set enrichment analysis (GSEA) of differentially expressed genes (DEGs) in small‐for‐gestational‐age 3 (SGA3) placenta compared with appropriate‐for‐gestational‐age (AGA) control. (A) GSEA reveals coordinated dysregulation of specific sets of genes between AGA and SGA3 placentas, highlighting alterations in lipid and hormone‐specific pathways (including prostaglandin, pantothenate, eicosanoid, synthesis very long‐chain acyl‐CoA, arachidonic acid metabolism, oestrogen response) that are not evident from individual gene changes alone; (B) Bar plot showing the normalised enrichment score (NES) for each significantly enriched pathway. Positive NES means the genes of the pathway are upregulated in SGA3 compared to AGA, negative NES means downregulated genes. Notably, the oestrogen pathway is enriched in SGA3 placentas, whereas pathways involving eicosanoid synthesis, arachidonic acid metabolism, very long‐chain acyl‐CoA synthesis and prostaglandin signalling are more enriched in AGA placentas.

To detect coordinated expression changes beyond individual DEGs, we performed gene set enrichment analysis (GSEA). This revealed that five gene sets related to lipid metabolism were significantly enriched in AGA placentas compared to SGA3 placentas, all with positive normalised enrichment scores (NES) between 1.39 and 1.97: prostaglandin signalling (*P* = 0.0002), pantothenate‐CoA biosynthesis (*P* = 0.008), eicosanoid synthesis pathway (*P* = 0.048), synthesis of very long‐chain fatty acyl‐CoA (*P* = 0.04) and arachidonic acid metabolism (*P* = 0.047). In contrast, the group of Hallmark oestrogen response genes was significantly enriched in SGA3 placenta (NES <0, *P* < 0.0001) (Fig. 1A,B). The enrichment of oestrogen response genes in SGA3 likely reflects a compensatory reaction to impaired steroid metabolism. Together, GO and GSEA analyses suggest impairment of normal steroid and lipid metabolism and activation of hypoxia and stress pathways in SGA3 placenta.

To complement the transcriptomic data, we performed untargeted metabolomic analysis on AGA and SGA3 placenta. Three SGA3 and three AGA placenta biopsies were subjected to LC/MS analysis and 1165 metabolites were quantified [16]. Of these, 19.4% (226 metabolites) were differentially accumulated metabolites (DAMs): 74 more accumulated and 152 less accumulated in SGA3 compared to AGA. GSEA of more accumulated DAMs revealed an overrepresentation of nicotinate and nicotinamide metabolism (*P* = 7.24 × 10^−4^), glycerophospholipid metabolism (*P* = 9.5 × 10^−3^) and arginine and proline metabolism (*P* = 1.4 × 10^−2^). Conversely, less accumulated DAMs were enriched in Steroid biosynthesis (*P* = 2.79 × 10^−4^), taurine and hypotaurine metabolism (*P* = 1.10 × 10^−2^) and glutathione metabolism (*P* = 3.2 × 10^−2^) [16]. These metabolomic changes are consistent with the transcriptomic data and suggest loss of normal steroid and lipid metabolism, activation of hypoxia and stress pathways, and energy adaptation in SGA3 placenta.

In summary, integrative transcriptomic and metabolomic analyses indicate that SGA3 placenta exhibits a stress‐adapted phenotype characterised by: (i) reduced steroid hormone biosynthesis and lipid metabolism; (ii) increased hypoxia and oestrogen response pathways; and (iii) changes in arachidonic acid and eicosanoid signalling, NAD/glutathione metabolism and arginine metabolism. Taken together, these alterations indicate a placenta struggling to maintain endocrine and metabolic homeostasis under growth‐restricted conditions.

### Cholesterol pathway differences between SGA and AGA placentas: Dramatic upregulation of *SCARB1* (HDL SR‐BI receptor) in SGA

As GO and GSEA indicated perturbations in lipid and steroid metabolism in SGA3 placentas, we asked whether the expression of genes involved in cholesterol (Chl) synthesis and transport into trophoblasts differs between AGA and SGA term placentas. Syncytiotrophoblasts (STBs) form a multinucleated epithelial layer covering the outer surface of chorionic villi and serve as the primary site for placental steroidogenesis, a process that requires Chl as a substrate [17, 18]. During gestation, the intrinsic capacity of STBs to synthesise Chl progressively diminishes; by the third trimester, steroid hormone production should depend largely on maternal Chl uptake from the circulation [17, 19, 20, 21].

**Fig. 2:**
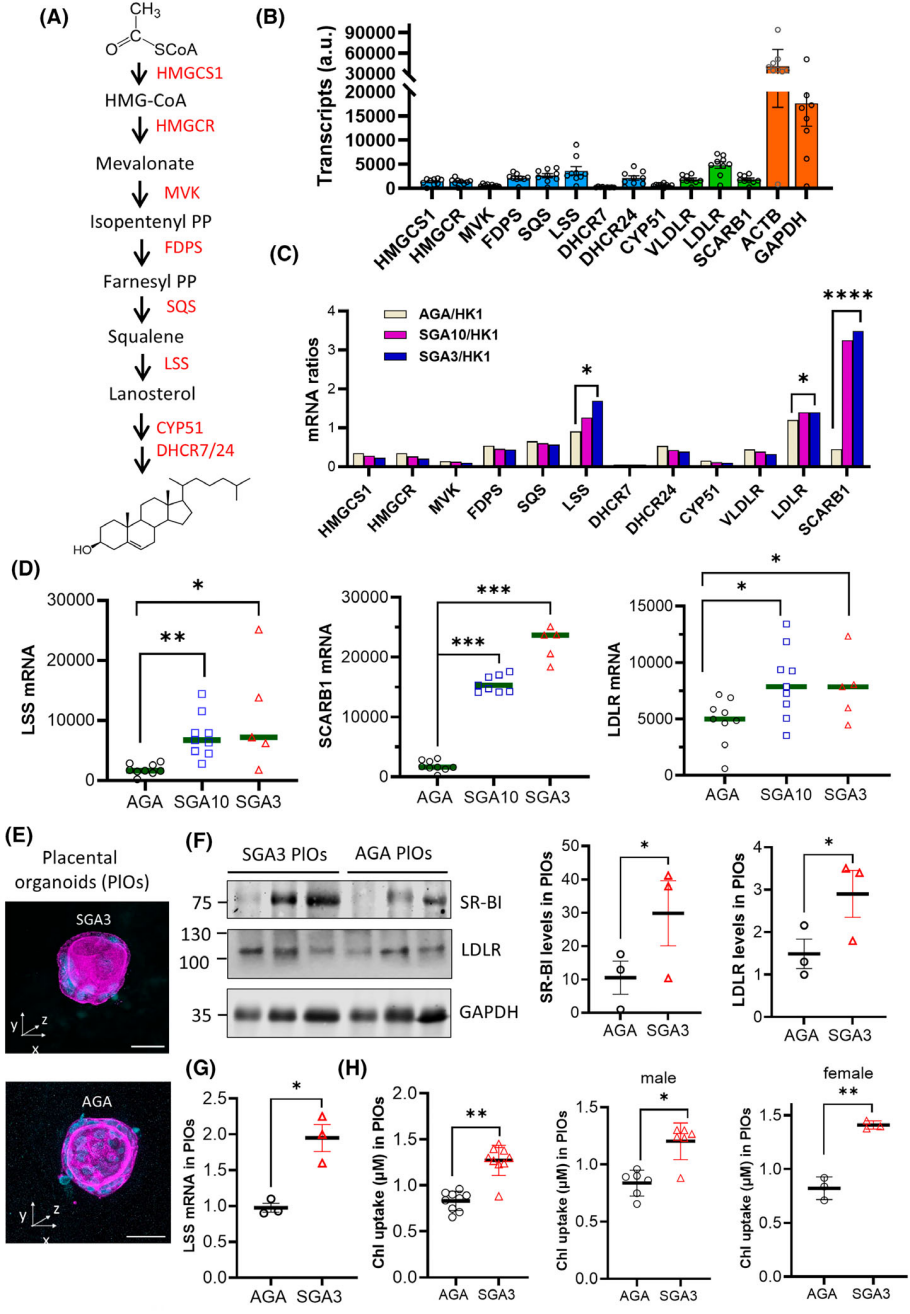
Expression of cholesterol (Chl) pathway enzymes and receptors in appropriate‐for‐gestational‐age (AGA) and small‐for‐gestational‐age (SGA) placental biopsies and organoids. (A) Schematic representation of the cholesterol (Chl) biosynthesis pathway. Names in red indicate enzymes, in black the metabolites; (B) Transcription levels of the main genes involved in the biosynthesis and transport of Chl in placenta. The housekeeping genes *ACTB* and *GAPDH* are reported for comparison. Data are from RNA‐seq performed on AGA placentas biopsies. Error bars represent ± SE (*n* = 9); (C) Expression levels of genes encoding key enzymes of the biosynthesis and transport of Chl in AGA (*n* = 9), SGA10 (*n* = 9) and SGA3 (*n* = 5) placental biopsies. The *SCARB1* is strongly upregulated in SGA placentas; (D) Transcript levels of *LSS*, *LDLR* and *SCARB1* in AGA (*n* = 9), SGA10 (*n* = 9) and SGA3 (*n* = 5) placental biopsies. For statistical analysis, the one sample t and Wilcoxon test were applied. *P*‐values ≤0.05 (*); 0.01 (**); 0.001 (***); (E) Confocal images of typical placental organoids obtained from AGA and SGA3 biopsies. Scale bar, 30 μm; (F) western blot showing the expression of the SR‐B1 and LDLR receptors in AGA and SGA3 placental organoids. The plots on the right show the levels of SR‐B1 and LDLR in the two types of placental organoids (PlOs). Error bars represent ± SE (*n* = 3); (G) level of *LSS* mRNA in AGA and SGA3 placental organoids. Error bars represent ± SE (*n* = 3); (H) Uptake of Chl in AGA and SGA3 PlOs. The data have been stratified between male and female placentas. Error bars represent ± SE (*n* = 9). The t‐test was applied. *P*‐values ≤ 0.05 (*); 0.01 (**); 0.001 (***).

To assess the Chl biosynthetic capacity of AGA placenta, we quantified the expression of genes in the Chl biosynthesis pathway relative to housekeeping genes such as glyceraldehyde 3‐phosphate dehydrogenase (GAPDH) and beta‐actin (ACTB) (Fig. 2A,B). Expression of the Chl pathway genes was markedly lower than that of housekeeping genes, suggesting that Chl biosynthesis is indeed minimal in third‐trimester placenta. This supports the notion that term placental steroidogenesis relies predominantly on exogenous, maternally derived Chl. Specifically, transcripts encoding 3‐hydroxy‐3‐methylglutaryl‐CoA reductase (HMGCR, the rate‐limiting enzyme of the pathway), 3‐hydroxy‐3‐methylglutaryl‐CoA synthase 1 (HMGCS1), mevalonate kinase (MVK), 7‐dehydrocholesterol reductase (DHCR7), 24‐dehydrocholesterol reductase (DHCR24) and cytochrome P450 51A1 (CYP51) were expressed at levels approximately 10‐ to 25‐fold lower than GAPDH and ACTB.

We then compared the expression of the Chl pathway genes between SGA and AGA placentas. Fig. 2C shows that the levels of most transcripts, normalised by the housekeeping HK1 gene, were comparable across groups except for three genes displaying significant differences: scavenger receptor class B member 1 (SCARB1), encoding the HDL‐Ch receptor SR‐BI [20, 22]; low‐density lipoprotein receptor (LDLR), encoding the LDL‐Ch receptor [23]; and lanosterol synthase (LSS), which catalyses the first committed step in sterol biosynthesis [24]. All three were significantly upregulated in SGA placentas (SGA10 and SGA3) relative to AGA. SCARB1 expression was elevated approximately ninefold in SGA10 and 14‐fold in SGA3, while LSS was upregulated about fivefold in SGA10 and sixfold in SGA3. LDLR expression showed a lower (about 1.5‐fold), yet significant, increase in SGA groups (Fig. 2D). The biological implication of enhanced lanosterol synthase expression remains unclear; however, lanosterol derivatives possess free radical‐scavenging activity, despite not being classical antioxidants such as vitamin C or glutathione. This property could help counteract the heightened oxidative stress commonly observed in SGA placentas [16, 25].

To validate these transcriptomic findings, we used 3D placental organoids (PlOs) generated from the AGA and SGA3 biopsies (Fig. 2E) [16, 26]. The trophoblast composition of SGA3 PlOs was previously determined and consisted of approximately 15% extravillous trophoblasts (EVTs), 30% STBs, and 55% cytotrophoblasts (CTBs) [16]. These cell populations were identified using established markers: TP63, XRCC6, and SRSF2 for CTBs; CGA, PSG2 and HSD3B1 for STBs; and MMP2, ITGA5 and FSTL3 for EVTs [16]. All PlOs secreted β‐human chorionic gonadotropin (β‐hCG), as expected [16]. Notably, while the trophoblast populations expanded efficiently in culture, the SGA3 PlOs exhibited reduced growth and expansion compared to AGA PlOs, consistent with impaired developmental potential. Placental organoids (PlOs) remain partially dedifferentiated in culture when maintained in TOM medium, adopting a pseudospherical configuration. In contrast, they invade the extracellular matrix when induced to transition towards EVT. Western blot experiments confirmed that the expression of the SR‐BI and LDLR receptors was approximately three‐ and twofold, respectively, higher in SGA3 PlOs compared to AGA PlOs (Fig. 2F). Similarly, RT‐qPCR analysis revealed a twofold increase in LSS mRNA expression in SGA3 PlOs (Fig. 2G).

It is known that SR‐BI and LDLR are high‐affinity receptors for HDL‐Chl and LDL‐Chl particles and play a central role in mediating the transfer of maternal Chl into the placenta [27]. To directly assess Chl uptake capacity in AGA and SGA3 PlOs, we used a fluorescent cholesterol analogue, nitrobenzoxadiazole‐conjugated cholesterol (NBD‐Chl). This probe is well established for monitoring lipoprotein receptor‐mediated Chl transport, as both SR‐BI and LDLR can internalise either NBD‐Chl alone or NBD‐Chl incorporated into HDL and LDL lipoproteins, respectively [28]. PlOs were incubated with NBD‐Chl for 20 h, after which intracellular fluorescence was quantified as a measure of Chl uptake. As shown in Fig. 2H, SGA3 PlOs exhibited approximately twofold higher NBD‐Chl uptake than AGA PlOs, consistent with our transcriptome (RNA‐seq of placental biopsies) and western blot data (placental organoids) showing upregulated SR‐BI and LDLR expression in SGA3 placentas. These findings strongly suggest that enhanced receptor expression in SGA3 is coupled to increased functional Chl uptake capacity, likely representing a compensatory mechanism to secure adequate Chl supply under conditions of placental insufficiency. When uptake was stratified by fetal sex, no significant differences were observed between male‐ and female‐derived PlOs, indicating that Chl transport via SR‐BI/LDLR is independent of fetal sex in the SGA context (Fig. 2H).

Collectively, these findings provide evidence that placental cholesterol receptors SR‐BI and, to a lesser extent, LDLR are consistently upregulated in SGA3 term placentas and their derived organoids. This upregulation likely reflects a compensatory adaptation aimed at enhancing maternal Chl uptake and sustaining steroid hormone production under conditions of placental dysfunction. Importantly, SCARB1 and LDLR could serve as potential biomarkers of placental adaptive responses to certain pregnancy complications.

### SGA placentas exhibit significant changes in steroidogenesis gene expression relative to AGA

**Fig. 3:**
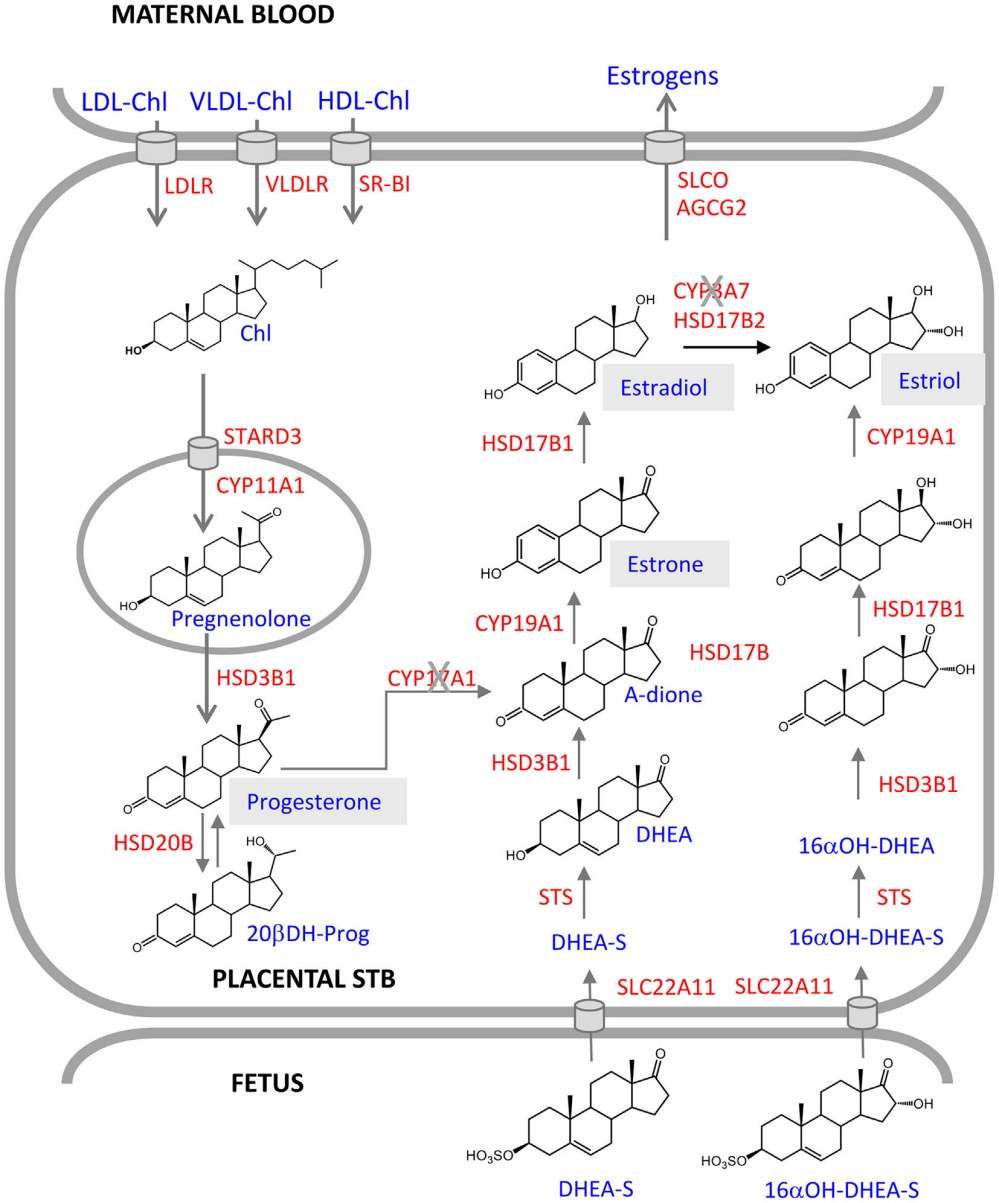
Steroidogenesis in the syncytiotrophoblast epithelium. Names in blue indicate metabolites, in red the enzymes. The CYP17A1 and CYP3A7 enzymes are not expressed in placenta. LDL‐Chl, VLDL‐Chl and HDL‐Chl represent cholesterol (Chl) associated with low‐density, very‐low‐density and high‐density lipoproteins, respectively. STB denotes the syncytiotrophoblast.

Although STBs are the primary source of progesterone and oestrogens during pregnancy, they lack CYP17A1, which is required for oestrogen biosynthesis. Consequently, placental steroidogenesis depends on precursors supplied by both the mother and the fetus [17]. This interdependence reflects the complementary enzymatic activities of the placenta and fetus within the feto‐placental steroidogenic unit (Fig. 3).

Two major enzyme classes drive placental steroidogenesis: (i) Cytochrome P450 (CYP) enzymes, localised in the inner mitochondrial membrane and endoplasmic reticulum, catalyse hydroxylation reactions using oxygen and NADPH [29, 30]; (ii) Hydroxysteroid dehydrogenases (HSDs), which utilise NAD(P)H and NAD(P)^+^ to interconvert hydroxysteroids and ketosteroids [31]. The absence of specific steroidogenic enzymes aligns with a recent study describing a placental transcriptomic void comprising 762 depleted transcripts [32]. Consistent with this, our RNA‐seq analysis revealed that several key steroidogenic genes—including cytochrome P450 1A1 (CYP1A1), cytochrome P450 17A1 (CYP17A1), cytochrome P450 11B1 (CYP11B1), cytochrome P450 21 (CYP21A2), sulfotransferase 2B1 (SULT2B1), aldo‐keto reductase 1C2 (AKR1C2), aldo‐keto reductase 1C3 (AKR1C3) and hydroxysteroid 17‐beta dehydrogenase 3 (HSD17B3)—were virtually undetectable in both AGA and SGA placentas. This transcriptomic deficiency most likely reflects the specialised endocrine role of the placenta and the fact that it is a temporary organ that relies on maternal physiology [33].

**Fig. 4:**
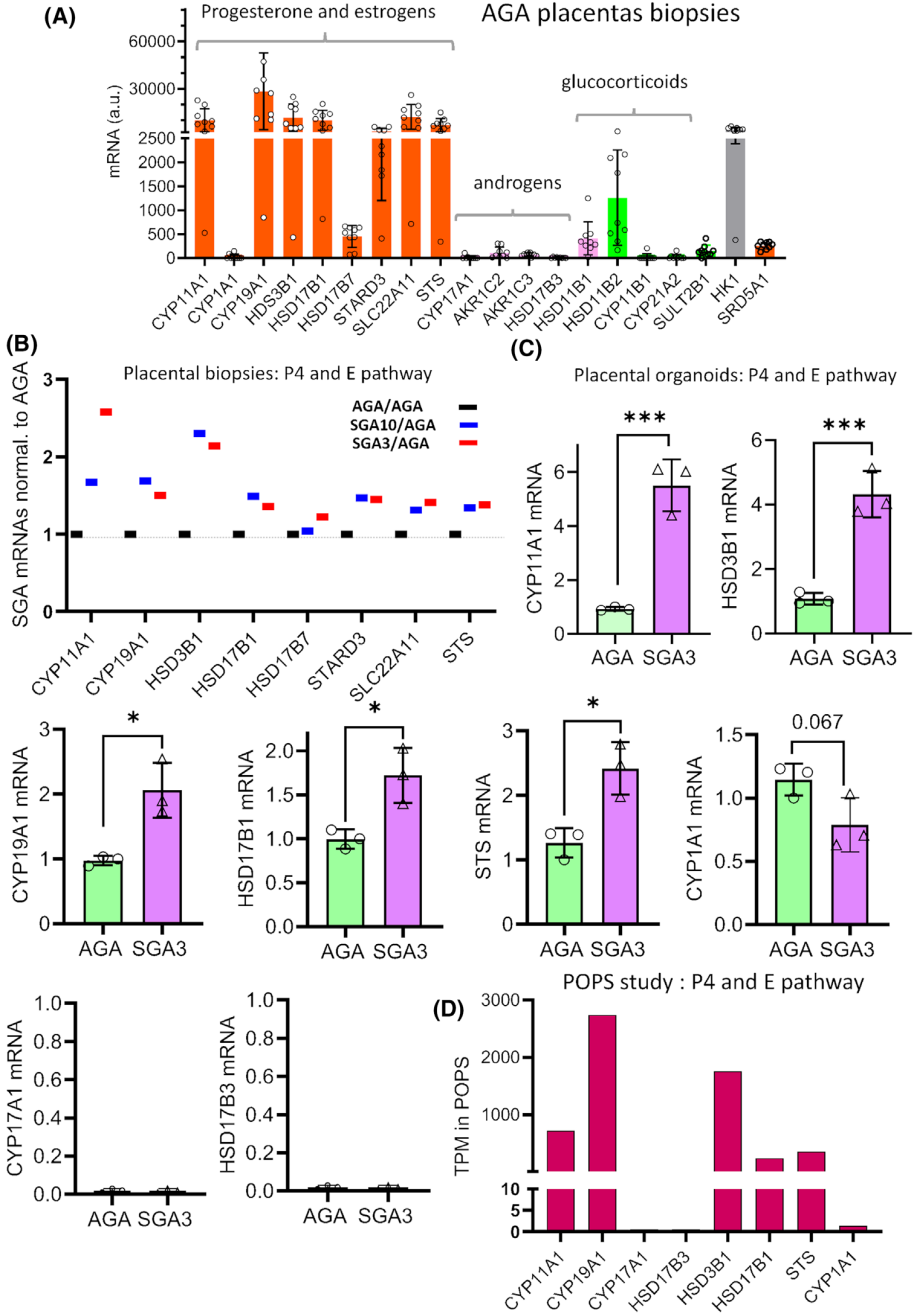
Expression of steroidogenic enzymes in appropriate‐for‐gestational‐age (AGA) and small‐for‐gestational‐age (SGA) placental biopsies and organoids. (A) Transcriptomic landscape of steroidogenesis in AGA placenta (*n* = 9) from RNA‐seq data performed on placental biopsies. The enzymes involved in the progesterone and oestrogen biosynthesis, androgens and glucocorticoids biosynthesis are grouped. The expression of the housekeeping gene HK1 is reported for comparison. Error bars represent ± SE (*n* = 9); (B) transcription levels of genes involved in the progesterone (P4) and oestrogen (E) pathways in SGA10 (*n* = 9) and SGA3 (*n* = 5) placental biopsies, normalised by the mean AGA (*n* = 9) expression; (C) levels of progesterone and oestrogen pathway enzymes determined by RT‐qPCR on RNA extracts from AGA and SGA3 placental organoids (PlOs). Error bars represent ± SD (*n* = 3). (D) Transcription levels expressed in TPM (transcripts per million) of genes involved in the progesterone and oestrogen pathway from the Pregnancy Outcome Prediction (POP) Study. The t‐test was applied. *P*‐values ≤ 0.05 (*); 0.001 (***).

As shown in Fig. 4A, expression profiles of steroidogenic genes in healthy AGA placentas indicate robust transcription of progesterone‐ and oestrogen‐related genes, whereas transcripts for androgen‐ and glucocorticoid‐associated pathways are low or absent. This expression pattern underscores the placenta's specialisation as an endocrine organ focused on progesterone and oestrogen production. Progesterone synthesis in STBs begins with the mitochondrial import of cholesterol, mediated by STARD3, followed by conversion to pregnenolone via CYP11A1, and subsequent transformation to progesterone by HSD3B1 in the endoplasmic reticulum [34, 35]. Progesterone is then transferred to the fetus and mother to sustain pregnancy [36]. Notably, our RNA‐seq data confirmed that CYP17A1—responsible for 17α‐hydroxylase activity—was absent in AGA biopsies (Fig. 4A). This enzymatic gap prevents the conversion of progesterone into androstenedione, the immediate oestrogen precursor, effectively restricting placental steroidogenesis. Consequently, oestrogen biosynthesis in STBs relies on fetal (and mother) adrenal precursors, such as dehydroepiandrosterone sulphate (DHEA‐S) and 16α‐hydroxy‐DHEA‐S, imported via SLC22A11 and desulphated by steroid sulfatase (STS). DHEA is then sequentially processed by HSD3B1, CYP19A1, and HSD17B1 to generate estrone (E1) and estradiol (E2) (Fig. 3). Owing to the absence of CYP3A7 in the placenta (confirmed by our RNA‐seq data and the Pregnancy Outcome Prediction Study (POPS) [37]), estriol (E3), the predominant pregnancy oestrogen, is synthesised through the 16α‐hydroxy‐DHEA pathway [38]. Functionally, estriol contributes to the feto‐placental unit by promoting vasodilation through stimulation of nitric oxide production [39]. Consistent with this, we previously reported that the endothelial nitric oxide synthase (NOS3) gene was upregulated in the SGA3 biopsies compared to AGA controls, and correspondingly, nitric oxide (NO) levels in SGA3 PlOs were higher than in AGA PlOs [16].

Importantly, we observed that SGA10 and SGA3 placentas expressed significantly higher levels of steroidogenic genes compared to AGA, particularly CYP11A1 and HSD3B1, which transform cholesterol into progesterone (Fig. 4B). This suggests an adaptive upregulation of progesterone synthesis under metabolic stress conditions. Similarly, increased expression of SLC22A11, STS, HSD3B1, HSD17B1 and CYP19A1 indicates enhanced utilisation of the DHEA‐S/16α and DHEA‐S in the oestrogen pathway in SGA placentas (Fig. 3).

To validate these critical findings, we assessed gene expression in both AGA and SGA3 PlOs. RT‐qPCR analysis confirmed a two‐ to fivefold upregulation of key steroidogenic genes in SGA3 PlOs compared with AGA controls (Fig. 4C). Notably, CYP11A1 and HSD3B1 were elevated by fivefold and 4.5‐fold, respectively, highlighting a marked shift towards increased progesterone synthesis in the SGA3 placenta. Similarly, transcripts encoding CYP19A1, HSD17B1 and STS, which are essential for oestrogen biosynthesis, were significantly higher in SGA3 PlOs than in AGA counterparts. Consistent with our biopsy‐based observations, CYP17A1 and HSD17B3 remained undetectable in both AGA and SGA3 PlOs, in line with the previously reported placental transcriptomic void [32]. Notably, RNA‐seq data from 295 biopsies in the Pregnancy Outcome Prediction Study (POPS) dataset [37] showed that expression of the steroidogenic genes closely matched those of AGA placentas shown in Fig. 4A, further supporting the robustness of our findings despite the limited size of the experimental samples (Fig. 4D).

**Fig. 5:**
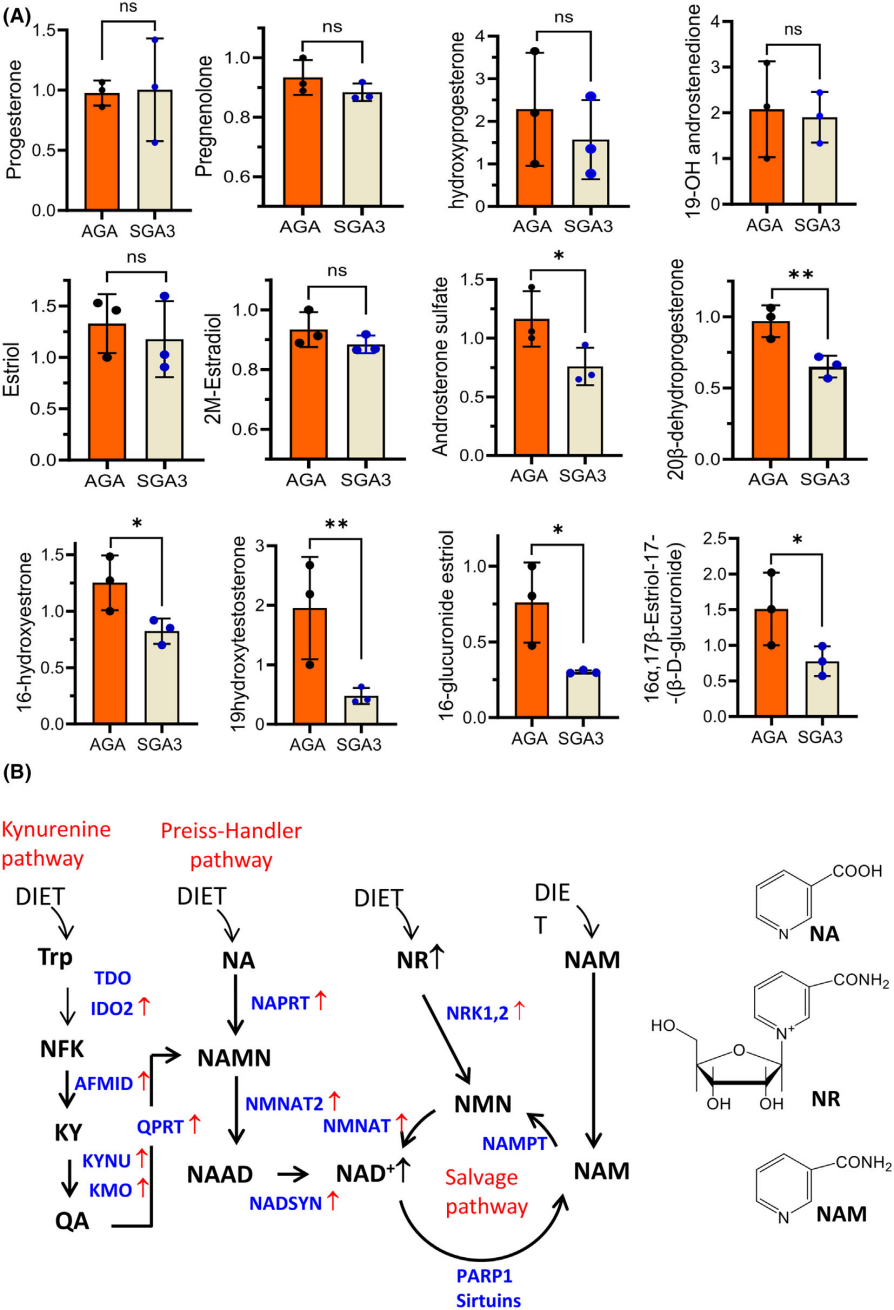
Metabolomic profiling of steroidogenic hormones in appropriate‐for‐gestational‐age (AGA) and small‐for‐gestational‐age (SGA) placental biopsies and NAD^+^ pathways. (A) Metabolomic data determined by LC/MS on AGA (*n* = 3) and SGA3 biopsies. Error bars represent ± SD (*n* = 3). For statistical analysis, the Student's *t*‐test was applied. *P*‐values ≤ 0.05 (*); 0.01 (**); ns, not significant; (B) Scheme showing the pathways for the synthesis of NAD^+^. The coenzyme is synthesised through the *de novo* synthesis, the Preiss–Handler pathway and the salvage pathway. The synthesis *de novo* starts with Trp while the Preiss–Handler pathway starts with nicotinic acid (NA) from dietary source which is converted into nicotinic acid mononucleotide (NAMN), nicotinic acid adenine dinucleotide (NAAD) and finally into NAD^+^. The salvage pathway uses nicotinamide riboside (NR) and nicotinamide (NAM), the product of NAD^+^ dependent enzymes such as PARP1 and sirtuins, to regenerate NAD+ via the intermediate nicotinamide mononucleotide (NMN). Enzymes with the vertical arrow are more expressed in SGA placenta than in AGA control. The other metabolites involved in the pathways are: N‐formil kynurenine (NFK), kynurenine (KY), quinolinic acid (QA), nicotinic acid mononucleotide (NAMN), nicotinic acid dinucleotide (NAAD), nicotinamide dinucleotide (NAD^+^) and nicotinamide mononucleotide (NMN).

Complementary metabolomic analysis of AGA and SGA3 placentas revealed that, while major steroids—including progesterone, pregnenolone, hydroxyprogesterone, 19‐hydroxyandrostenedione and estriol—were maintained at comparable levels, several downstream metabolites were significantly reduced in SGA3 (Fig. 5A). These included androsterone sulphate, 20β‐dehydroprogesterone, 16‐hydroxyestrone, 19‐hydroxytestosterone, estriol‐16‐glucuronide and 16α,17β‐estriol‐(β‐D‐glucuronide). Thus, despite placental impairment, primary steroidogenesis in SGA3 placenta remains functionally intact, likely maintained by compensatory transcriptional upregulation of key enzymes. However, the depletion of hydroxylated, reduced and conjugated steroid metabolites indicates dysregulated downstream metabolic processing, rather than a defect in the core steroidogenic pathways.

Together, these findings show that SGA placentas mount a compensatory steroidogenic response, upregulating progesterone and oestrogen synthesis genes and thereby preserving primary steroid levels. Nonetheless, secondary metabolism of steroid hormones is impaired, suggesting selective vulnerability of downstream enzymatic processes to placental stress.

### In the SGA3 placenta, the NAD ^+^‐salvage pathway is upregulated to maintain steroidogenesis

The healthy AGA placenta functions as a highly efficient organ that optimises the exchange of nutrients and oxygen, maintains redox balance, and supports fetal growth. In contrast, the SGA3 placenta acts as a stressed and metabolically restricted organ. Gene Ontology (GO) analysis of downregulated DEGs in the SGA3 placenta reveals impaired metabolic efficiency and increased oxidative stress, mainly due to chronic hypoxia. To counteract the excessive accumulation of reactive oxygen species (ROS) and sustain steroidogenesis and energy production under stress conditions, the SGA3 placenta activates compensatory responses along two main regulatory axes.

**Fig. 6:**
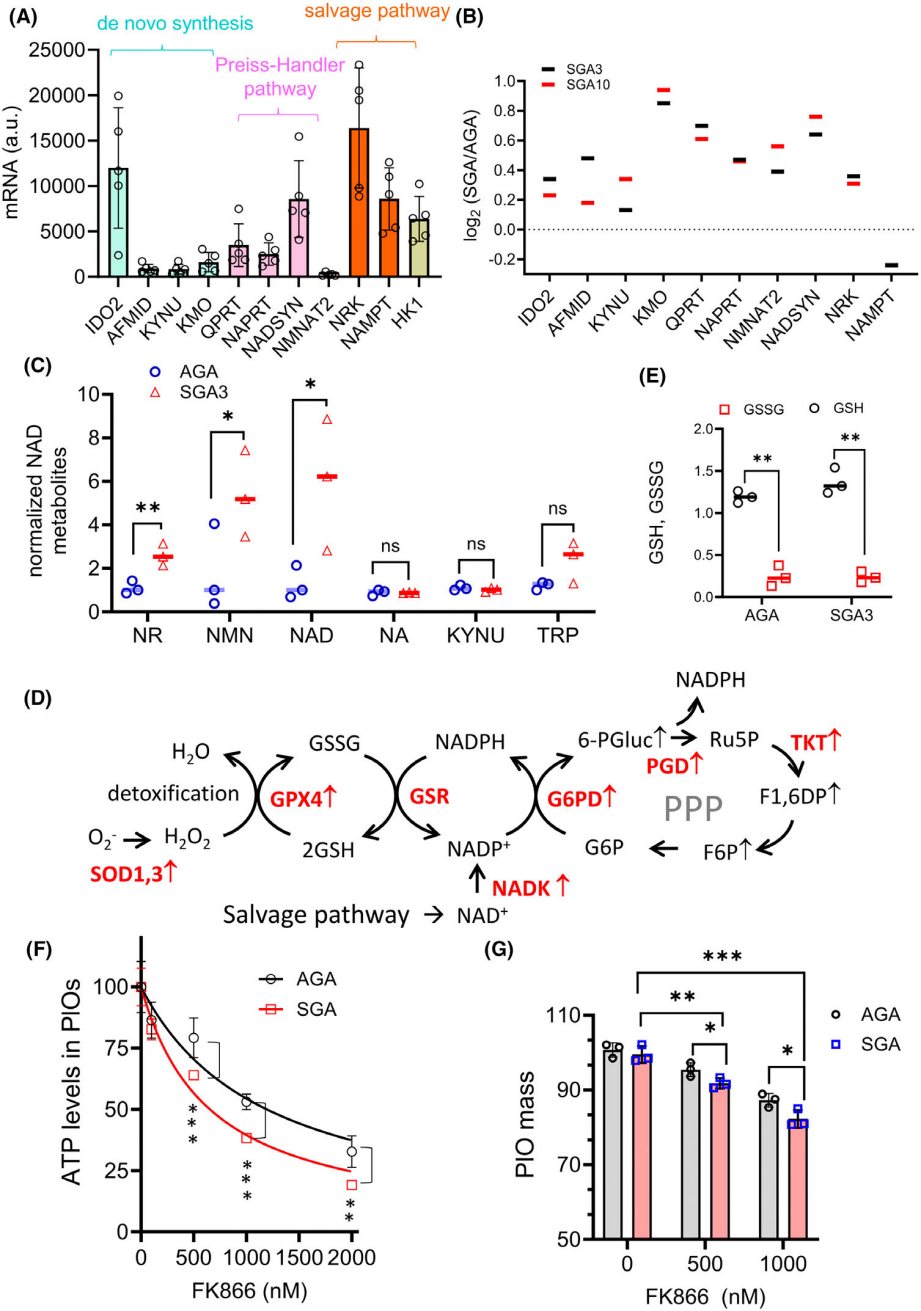
Enzymatic and metabolomic profiling of NAD pathways in appropriate‐for‐gestational‐age (AGA) and small‐for‐gestational‐age (SGA) placental biopsies. (A) Transcript abundance of genes involved in NAD^+^ metabolism (*de novo* biosynthesis, Preiss–Handler pathway, salvage pathway) in SGA3 placenta. (B) log_2_ (mRNA_SGA_/mRNA_AGA_) of NAD^+^ genes in SGA3 and SGA10 placentas compared to AGA control. All enzymes except NAMPT are more expressed in SGA placentas than in AGA; (C) levels of metabolites in SGA3 biopsies, normalised by AGA value, determined by LC/MS; (D) The glutathione‐NAPH‐PPP metabolic scheme showing the reduction of NADP^+^ into NADPH via the pentose phosphate pathway (PPP) and its detoxification function via the glutathione cycle in placenta; (E) levels of glutathione reduced GSH and oxidised GSSG in placental AGA and SGA3 biopsies. The reduced form is significantly higher than the oxidise form both in AGA and SGA3 placentas; (F) levels of ATP in AGA and SGA3 placental organoids (PlOs) as a function of FK866, an inhibitor of the salvage pathway; (G) mass of AGA and SGA3 PlOs as a function of FK866. Error bars in (A), (F) and (G) represent ± SD (*n* = 3). Statistical analysis was performed using the Student's *t*‐test. *P*‐values ≤ 0.05 (*); 0.01 (**); 0.001 (***); ns = not significant.

The first compensatory mechanism involves replenishment of NAD^+^ through the salvage pathway (Fig. 5B). Consistent with this, GSEA of differentially accumulated metabolites revealed that Nicotinate and nicotinamide metabolism is one of the most enriched metabolic pathways, emphasising the central role of NAD metabolism in the adaptive landscape. NAD^+^ can be generated in three ways: (i) *de novo* synthesis from tryptophan [40], (ii) the Preiss–Handler pathway using dietary nicotinic acid (NA) or (iii) the salvage pathway that recycles dietary nicotinamide riboside (NR) and nicotinamide (NAM) generated by NAD^+^‐consuming enzymes such as PARPs and sirtuins [41, 42, 43] (Fig. 5B). Expression analyses revealed that the key salvage pathway enzymes NRK and NAMPT are highly expressed in SGA3 placentas (Fig. 6A). Furthermore, with the exception of NAMPT, all enzymes in this pathway showed significantly higher expression in SGA3 compared to AGA placentas (Fig. 6B). These data suggest a preferential activation of the salvage pathway under oxidative stress and nutrient restriction. Metabolomic profiling supports this shift: NR, NMN and NAD^+^ are significantly more abundant in SGA3 biopsies, while precursors for NAD^+^ biosynthesis (nicotinic acid, L‐kynurenine and tryptophan) remain comparable between AGA and SGA3 (Fig. 6C).

The upregulation of steroidogenic enzymes in SGA3 placenta (Fig. 4B,C) places additional demands on the NAD pool, which is already stressed by the need for antioxidants. Thus, the placenta is under double metabolic pressure to maintain (i) redox homeostasis through NADPH‐driven antioxidant systems (e.g. glutathione recycling) and (ii) steroidogenesis, which requires NAD‐dependent dehydrogenases and NADPH‐dependent cytochrome P450 oxidoreductases. This competition likely creates a metabolic trade‐off: resources used for the biosynthesis of steroid hormones can limit ROS detoxification, thereby maintaining oxidative stress in SGA3 placenta despite robust compensatory mechanisms [16]. Several NAD‐dependent steroidogenic enzymes are involved in steroidogenesis, including: (i) NAD^+^‐dependent dehydrogenases: 3β‐HSD (pregnenolone → progesterone; DHEA → androstenedione), 17β‐HSD (estrone → estradiol; androstenedione → testosterone) and 20α‐HSD (progesterone → 20α‐hydroxyprogesterone) [30]; (ii) cytochrome P450 enzymes: CYP11A1, CYP17A1 and CYP19A1, which are dependent on NADPH [29].

The second compensatory mechanism involves the coordinated action of the glutathione (GSH) cycle and the pentose phosphate pathway (PPP) (Fig. 6D). SGA placentas show marked upregulation of genes involved in glutathione recycling, a process that neutralises H_2_O_2_ and lipid peroxides. Key genes include SOD3 (upregulated by ~42%, *P* < 0.05) and GPX4 (upregulated by 20%, *P* < 0.01). This detoxification requires NADPH, which is supplied by the cooperation of the NAD^+^ salvage pathway and PPP. Mechanistically, NAD^+^ recycled via the salvage pathway is phosphorylated by NADK (upregulated by ~40% in SGA, *P* < 0.01) to produce NADP^+^. This is then reduced to NADPH through the PPP, a process involving upregulated enzymes TKT (~37%, *P* < 0.05) and PGD (~15%, *P* < 0.05) in SGA (Fig. 6D) [16]. This coordinated system fuels antioxidant defences, ensuring that the glutathione pool remains predominantly in its reduced form in both AGA and SGA placentas (Fig. 6E).

To directly test the role of the salvage pathway, we treated AGA and SGA3 organoids with FK866, a potent and specific inhibitor of NAMPT, the rate‐limiting enzyme of this pathway [44]. FK866 depletes intracellular NAD^+^ and thereby impairs NAD^+^‐dependent processes. Because SGA3 organoids are highly dependent on glycolysis [16], and thus on NAD^+^, we predicted a greater reduction in ATP production upon treatment with FK866 compared to AGA controls. Consistent with this, FK866 reduced ATP content in both organoid types in a dose‐dependent manner, but with a significantly greater effect in SGA3 (Fig. 6F). We also measured the effect of FK866 on organoid mass and found that the NAMPT inhibitor at a dose of 2 μm caused a 20% reduction in organoid mass (Fig. 6G).

In summary, activation of the NAD^+^‐salvage pathway is an important adaptive strategy in the SGA3 placenta. By replenishing the intracellular NAD^+^ pool, the pathway simultaneously supports antioxidant defence, maintains steroid hormone biosynthesis, preserves genome integrity and improves cell survival under the metabolic and oxidative stress of placental insufficiency.

### Deficiency of ω‐3 and ω‐6 PUFAs, lysophospholipids and eicosanoids in the SGA placenta

Metabolomic profiling of placental biopsies revealed a significant deficiency of several lipid classes in SGA3 placentas compared with AGA controls, including polyunsaturated fatty acids (PUFAs), lysophospholipids (LPLs) and monoacylglycerols (MGs) (Tables 1 and 2). These lipids act as reservoirs for eicosanoid biosynthesis and for PUFAs essential to the fetus. Eicosanoids are a diverse family of bioactive lipid mediators indispensable for placental homeostasis, regulating vascular tone, maternal–fetal nutrient transfer and immune tolerance [45, 46].

**Table 1:** Critical ω‐3 and ω‐6 fatty acids are less abundant in SGA placenta than in AGA control.

Fatty acids FA^a^	Type of FA	Lower in SGA3	*P*‐value	Name
20:4	ω‐6	YES	0.007	ARA, Arachidonic acid
20:3	ω‐6 (Δ8,11,14)	YES	0.005	DGLA, Dihomo‐γ‐linolenic acid
22:4	ω‐6	YES	0.036	Adrenic acid
16:2n‐6	ω‐6	YES	0.019	3‐hydroxyhexadecanoid acid
20:3n‐6	ω‐6	YES	0.0051	5,8,11‐Eicosatrienoic acid
16:2 n‐6	ω‐6	NO	0.326	Hexadecadienoic acid
22:5	ω‐3	NO	0.140	DPA, docosapentaenoic acid
20:6	ω‐3	YES	0.057	EHA eicosahexaenoic acid
20:5	ω‐3	NO	0.089	EPA, eicosapentaenoic acid
22:6	ω‐3	YES	0.041	DHA, Docosahexaenoic acid
24:5n‐3	ω‐3	YES	0.050	Tetracosapentaenoic acid
12:1	ω‐3	NO	0.286	Lauroleic acid
15:0	Saturated	NO	0.233	Pentadecanoic acid
18:0	Saturated	NO	0.164	Stearic acid
21:0	Saturated	YES	0.006	Heneicosanoic acid
16:0	Saturated	YES	0.019	3‐Hydroxyhexadecanoic acid

^a^ Data obtained from LC/MS‐based metabolomic analyses of placental biopsies from AGA and SGA3 groups.

**Table 2:** Critical lysophospholipids and monoacylglycerols are less abundant in SGA placenta compared to AGA control (data from metabolomics). LPC, lysophosphatidylcholine; LPE, lysophosphatidylethanolamine; LPL, lysophospholipid; LPS, lysophosphatidylserine; MG, monoacylglycerol.

LPL^a^	Type of FA in LPLs and MGs	Lower in SGA3	*P*‐value
LPC (18:3/0:0)	ω‐3	YES	0.0174
LPC (0:0/18:2)	ω‐6	YES	0.0066
LPC (20:3/0:0)	ω‐6	YES	<0.05
LPC (18:1/0:0)	Neither ω‐6 nor ω‐3	YES	0.0487
LPC (0:0/22:6)	ω‐3	NO	>0.05
LPC (22:5/0:0)	ω‐3	NO	>0.05
LPC (0:0/22:4)	ω‐6	NO	>0.05
LPC (0:0/22:5)	ω‐3	NO	>0.05
LPE (18:2/0:0)	ω‐6	YES	0.0342
LPE (18:3/0:0)	ω‐3	YES	<0.05
LPE(0:0/18:0)	Neither ω‐6 nor ω‐3	YES	0.0237
LPE (20:1/0:0)	Neither ω‐6 nor ω‐3	YES	0.0319
LPE (22:5/0:0)	ω‐6	YES	0.0292
LPE (0:0/16:0)	Neither ω‐6 nor ω‐3	YES	0.0383
LPS (20:0)	Neither ω‐6 nor ω‐3	NO	>0.05
LPS (18:3)	ω‐3	NO	>0.05
MG (0:0/20:4/0:0)	ω‐6	YES	0.0303
MG (20:4/0:0/0:0)	ω‐6	YES	0.0387

^a^ Data obtained from LC/MS‐based metabolomic analyses of placental biopsies from AGA and SGA3 groups.

**Fig. 7:**
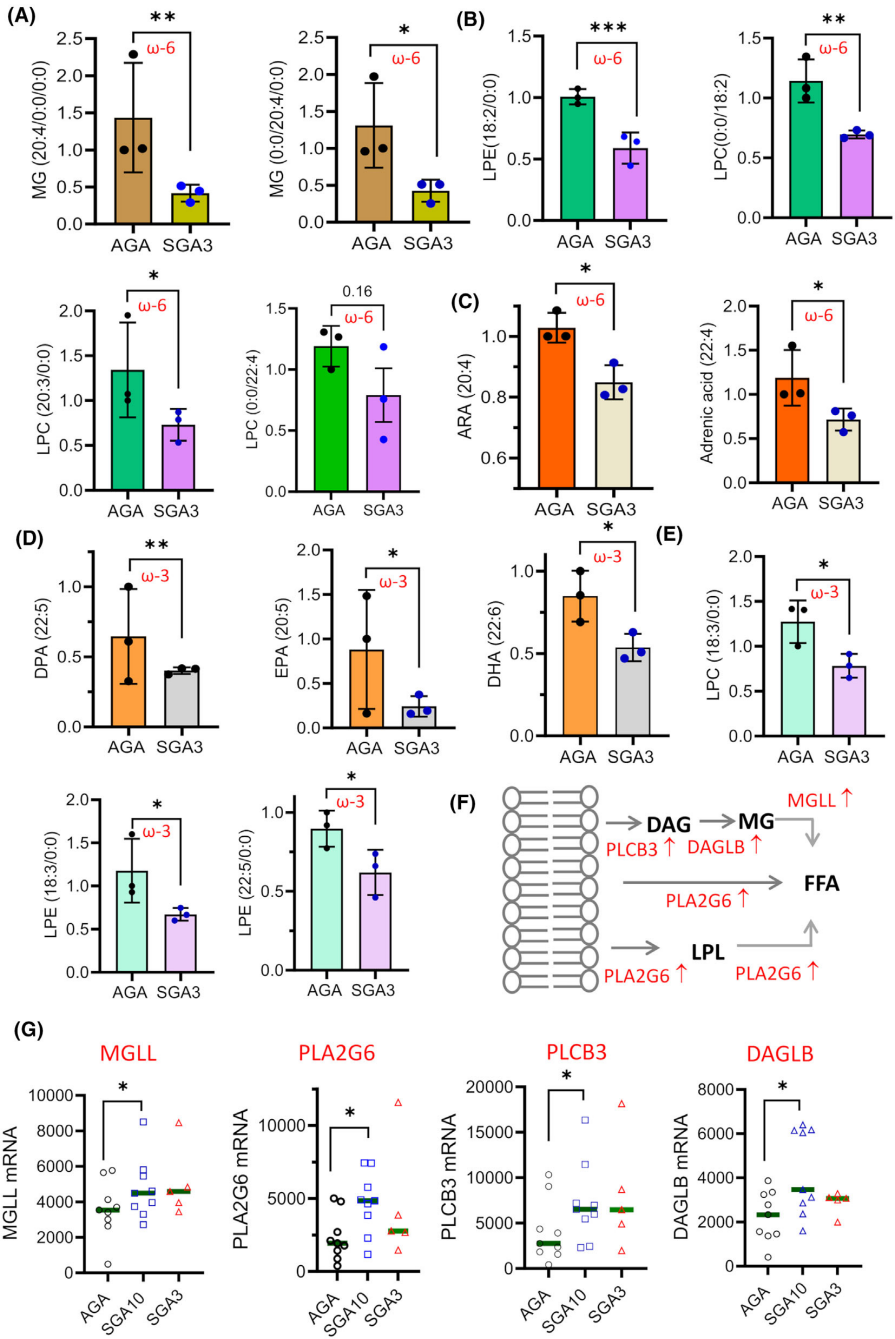
Metabolomic profiling of lipids in appropriate‐for‐gestational‐age (AGA) and small‐for‐gestational‐age (SGA) placental biopsies. (A, B) Metabolomic data showing the levels of monoacylglycerol (MG)‐ and lysophospholipid (LPL)‐containing ω‐6 FAs in AGA and SGA3 biopsies; (C) levels of ω‐6 fatty acids (FAs) in AGA and SGA placentas; (D) metabolomic data showing the levels of ω‐3 FAs in AGA and SGA placentas; (E) metabolomic data showing the levels of LPLs containing ω‐3 FAs in AGA and SGA placentas; (F) enzymatic scheme showing the release of fatty acids from the membrane phospholipids. Enzyme names are shown in red and metabolites in black; upward arrows indicate upregulated enzymes; (G) expression levels of the enzymes involved in the release of polyunsaturated (PUFAs) from RNA‐seq data. Error bars in (A), (B), (C), (D) and (E) represent ± SD (*n* = 3). Statistical analysis was performed using the one sample t and Wilcoxon test. *P*‐values ≤ 0.05 (*); 0.01 (**); 0.001 (***); LPE, lysophosphatidyletanolamine; LPC, lysophosphatidylcholine.

Among the most significantly decreased metabolites in SGA3 placentas were arachidonic acid (ARA)‐containing MG species, including MG(0:0/20:4/0:0) and MG(20:4/0:0/0:0) (Fig. 7A), as well as LPL‐containing ARA precursors, such as LPC(0:0/18:2), LPE(18:2/0:0), LPC(20:3/0:0) and LPC(0:0/22:4) (Fig. 7B, Table 2). These lipids can be hydrolysed by phospholipase PLA2G6 to release free ω‐6 fatty acids. The levels of ARA (20:4) and adrenic acid (22:4) were lower in SGA3 compared to AGA (Fig. 7C). The fact that the phospholipase PLA2G6 transcript is more abundant in SGA10 than in AGA placentas suggests an adaptive attempt to compensate for PUFA scarcity. However, despite this compensatory response, free ARA and adrenic acid remained reduced in SGA3 placentas compared to AGA. This reduction is likely due to their intensive utilisation in the biosynthesis of prostaglandins and thromboxanes, lipid mediators that play critical roles in trophoblast proliferation, invasion and uteroplacental vascular remodelling [45, 46].

The deficiency of lipids extended beyond ω‐6 lipids. Parallel reductions were observed in ω‐3 PUFAs, including docosapentaenoic acid (DPA, 22:5), eicosapentaenoic acid (EPA, 20:5) and docosahexaenoic acid (DHA, 22:6) (Table 1, Fig. 7D). Strikingly, ω‐3–enriched LPLs such as LPC(18:3/0:0), LPE(18:3/0:0), LPS(18:3/0:0) and LPE(22:5/0:0), precursors essential for DHA biosynthesis, were also deficient (Fig. 7E). Given DHA's central role in fetal neurogenesis, synaptogenesis and retinal development [47, 48], its depletion may affect long‐term neurodevelopmental outcomes in growth‐restricted offspring. The combined loss of DHA and EPA, both of which regulate inflammation, vascular integrity and neural maturation [47, 49], suggests an impairment of PUFA‐dependent signalling networks in the SGA placenta.

The lipid insufficiency appears to trigger compensatory transcriptional responses. Genes encoding lipid‐hydrolysing enzymes such as PLCB3 (phospholipase C beta 3), DAGLB (diacylglycerol lipase beta) and MGLL (monoacylglycerol lipase) were significantly upregulated (Fig. 7F,G). These enzymes mobilise fatty acids from lipid stores, reflecting an adaptive effort to replenish PUFA pools under nutrient‐limited conditions.

**Fig. 8:**
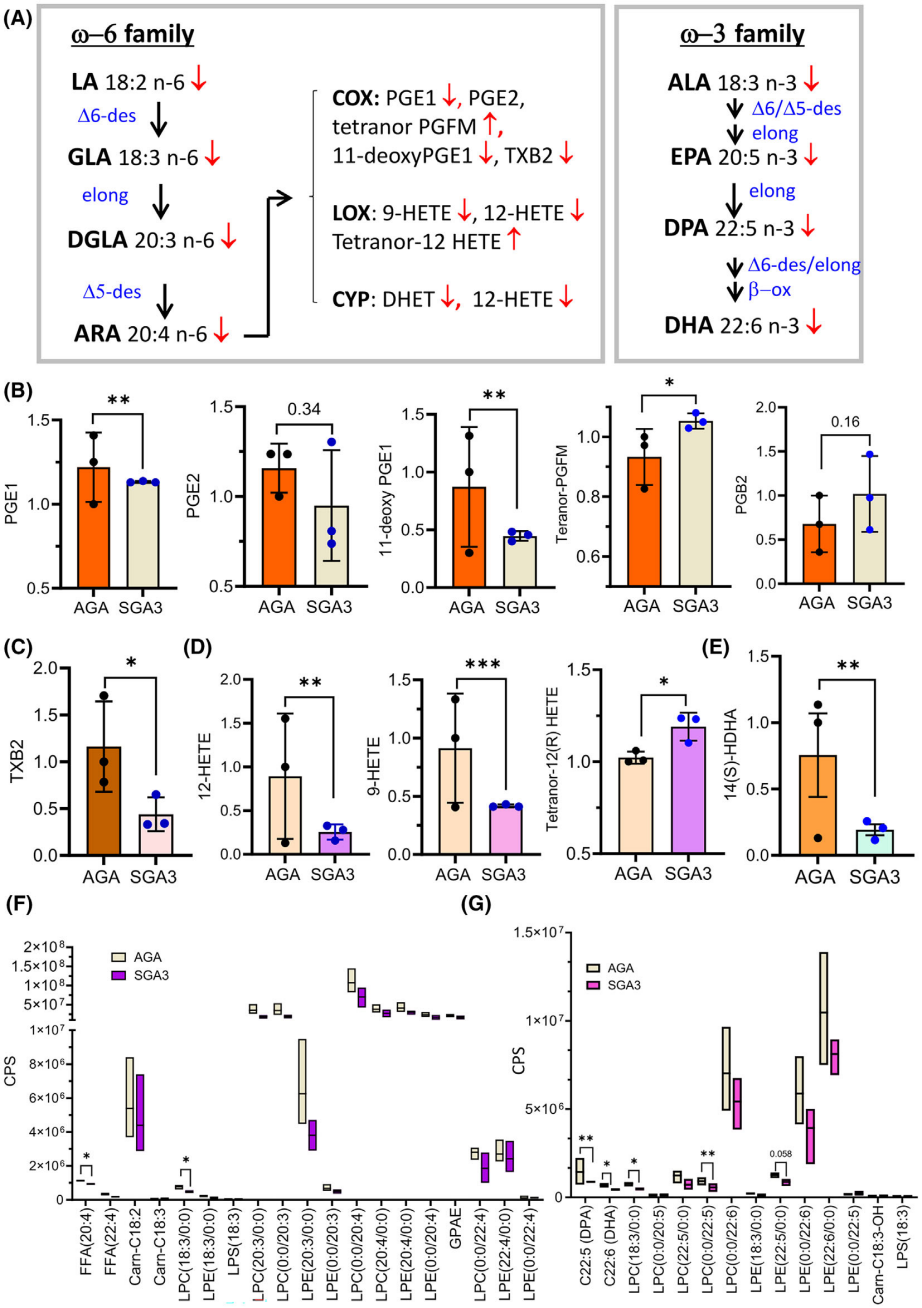
Lipidomic profiling of eicosanoids and lysophospholipids in appropriate‐for‐gestational‐age (AGA) and small‐for‐gestational‐age (SGA) placental biopsies. (A) The ω‐6 and ω‐3 families of long‐chain PUFAs. ALA, a‐linolenic acid; ARA, arachidonic acid; COX, cyclooxygenase; CYP, cytochrome P450 epoxygenase; DGLA, dihomo‐γ‐linolenic acid; DHA, docosahexaenoic acid; DPA, docosapentaenoic acid; EPA, eicosapentaenoic acid; GLA, γ‐linolenic acid; HDHA, hydroxydocosahexaenoic acid; HETE, hydroxyeicosatetraenoic acid; LA, linoleic acid; LOX, lipoxygenase; PG, prostaglandin; TX, thromboxane; (B–E) metabolomic data showing the levels of important eicosanoids in AGA and SGA placentas. Upward arrows indicate upregulated in SGA3, while downward arrows indicate downregulated in SGA3 compared to AGA; (F) Levels ω‐6 ARA and adrenic acid, and lysophospholipids containing ω‐6 FA in AGA and SGA3 placental biopsies. Data (counts per second CPS) are from LC/MS of AGA and SGA3 biopsies. GPAE, glycerophospho‐N‐arachidonoyl ethanolamine. Error bars represent ± SD (*n* = 3). Statistical analysis was performed using the one sample t and Wilcoxon test. *P*‐values ≤ 0.05 (*); 0.01 (**); 0.001 (***); (G) levels ω‐3 DPA and DHA, and lysophospholipids containing ω‐3 FA in AGA and SGA3 placental biopsies. Data (CPS) are from LC/MS of AGA and SGA3 biopsies. Statistical analysis was performed using the Student's *t*‐test. *P*‐values ≤ 0.05 (*); 0.01 (**).

The downstream consequences of reduced availability of ARA or its precursors were evident in the eicosanoid pathway (Fig. 8A). Levels of prostaglandins (e.g. 11‐deoxyPGE1, PGE1) and thromboxane B2 (TBX2) were significantly diminished in SGA3 placentas (Fig. 8A–C), along with oxidised ARA (12‐HETE and 9‐HETE) and DHA (HDHA) derivatives (Fig. 8D,E). These metabolites regulate placental haemodynamics, vascular remodelling and fetal oxygenation [50]. Their collective reduction points to a broad impairment of eicosanoid biosynthesis, which likely disrupts vascular adaptation and contributes to the pathophysiology of fetal growth restriction.

A deeper view of placental lipid remodelling was obtained by plotting the relative abundance of ω‐6 and ω‐3 free fatty acids and their lysophospholipid counterparts in SGA3 versus AGA placentas. As shown in Fig. 8F, free ω‐6 arachidonic (ARA, 20:4) and adrenic (22:4) acids were markedly depleted in SGA3. In contrast, ARA‐containing lysophospholipids (LPLs)—specifically LPCs and LPEs—and, to a lesser extent, adrenic acid‐containing LPLs accumulated to significantly higher levels. This pattern suggests enhanced activity of placental phospholipases PLA2G6, whose expression was indeed higher in SGA than in AGA placentas (Fig. 7G). The paradox of low free ARA but elevated ARA‐LPLs suggests that released fatty acids are rapidly consumed, likely funnelled into eicosanoid biosynthesis, while LPLs accumulate as a reservoir for future mobilisation and/or for export of ARA to the fetus. This enables regulated release of free ARA for eicosanoid synthesis, preventing an excess of free ARA that could trigger uncontrolled inflammation [51]. In addition, LPL‐PUFAs serve as a vehicle for supplying ARA and DHA to the fetus [52]. We observed that MSFD2A, which encodes a transporter for LPC‐PUFAs, is upregulated in SGA placenta (approximately 75%, *P* < 0.05). A similar lipid pattern was seen for ω‐3 fatty acids. Fig. 8G shows reduced free DHA but elevated DHA‐containing LPE and LPC species. The lysophospholipid form of DHA is more stable than the free fatty acid and can be efficiently transported to the fetus, whereas free DHA is prone to degradation via β‐oxidation. This highlights the central role of LPLs as both a buffer against PUFA depletion and a vehicle for fetal supply.

In summary, the SGA3 placenta exhibits a broad deficiency of key ω‐6 and ω‐3 PUFAs, their lysophospholipid precursors, and downstream eicosanoids. The resulting disruption of PUFA‐derived signalling cascades likely compromises antioxidant defences, vascular regulation and fetal neurodevelopment, thereby exacerbating the clinical burden of placental insufficiency.

## Discussion

This study demonstrates that metabolic perturbations occur in SGA placentas, including altered steroidogenesis, enhanced NAD^+^ salvage pathway activity, and significant depletion of PUFAs and eicosanoids. Building on previous evidence implicating arginine metabolism as a central adaptive pathway in growth restriction [16], our findings reveal additional strategies by which the placenta attempts to preserve its functions under adverse intrauterine conditions. The term placenta is a highly active steroidogenic organ but has limited capacity for *de novo* cholesterol (Chl) biosynthesis, making it dependent on maternal supply. Consistent with this, our transcriptomic data confirmed strong downregulation of key Chl biosynthetic genes in both AGA and SGA placentas, in agreement with the physiological downregulation of Chl biosynthesis in late gestation [53]. However, our RNA‐seq and immunoblotting revealed marked upregulation in SGA3 placentas of the genes encoding Chl uptake receptors: SCARB1 (>10‐fold increase by RNA‐seq) and LDLR (~1.5‐fold). This was mirrored at the protein level by elevated SR‐BI (~3‐fold) and LDLR (~2‐fold). These receptors facilitate the uptake of HDL‐Chl and LDL‐Chl from maternal circulation, underscoring the reliance of term placental steroidogenesis on maternal Chl and the coordinated maternal–fetal–placental unit. Functional studies using fluorescent NBD‐Chl confirmed enhanced uptake of Chl in SGA3 placental PlOs compared to AGA control organoids, consistent with receptor upregulation. Previous studies identified SCARB1/SR‐BI among genes upregulated in preeclamptic placentas [54, 55].

We also found that the steroidogenic enzymes CYP11A1, HSD3B1 and HSD17B1 were upregulated, particularly in SGA3 placentas, suggesting increased steroidogenic flux to maintain progesterone and oestrogen biosynthesis—hormones essential for sustaining pregnancy and supporting fetal growth [56]. The upregulation was confirmed in organoids derived from SGA3 and AGA placental biopsies, further substantiating this adaptive endocrine response. The role of CYP17A1 in placental steroidogenesis (Fig. 3) remains a subject of debate. Although *in vitro* studies using JEG‐3 cells and primary trophoblast cultures have reported detectable CYP17A1 expression [57, 58], our RNA‐seq analysis of 23 placentas (9 AGA and 14 SGA) and their corresponding placental organoids revealed a complete absence of CYP17A1 transcripts. This finding aligns with data from the Human Protein Atlas [59], the Pregnancy Outcome Prediction Study (POPS) dataset [37], and early biochemical reports demonstrating minimal or undetectable CYP17A1 activity *in vivo* [60]. Such discrepancies between *in vitro* and *in vivo* findings may reflect epigenetic silencing mechanisms, post‐transcriptional regulation or the absence of critical microenvironmental and endocrine cues in culture models necessary for CYP17A1 regulation. Functionally, the absence of CYP17A1 in the placenta indicates that progesterone, not being converted into androstenedione, cannot serve as an oestrogen precursor. Instead, placental oestrogen biosynthesis relies on the uptake of dehydroepiandrosterone sulphate (DHEA‐S) and 16α‐hydroxy‐DHEA‐S derived from the fetal (and mother) adrenal glands, which are subsequently metabolised within the syncytiotrophoblast to produce oestrogens (Fig. 3).

A second major adaptation was identified in NAD^+^ metabolism. Metabolomic profiling revealed a ~6‐fold elevation of NAD^+^ in SGA3 placentas, accompanied by increased levels of its salvage precursors, nicotinamide riboside (NR, 2.5‐fold) and nicotinamide mononucleotide (NMN, 3‐fold) (Fig. 6C). In contrast, levels of tryptophan, kynurenine, N‐formyl‐kynurenine and nicotinic acid (NA) were unchanged between groups (Fig. 6C), suggesting preferential activation of the salvage pathway rather than *de novo* or Preiss–Handler routes. This was reinforced by high expression of NRK and NAMPT, which catalyse NR and NAM utilisation (Fig. 6A). Notably, all enzymes in the NAD^+^ biosynthetic network, except NAMPT, were expressed more strongly in SGA3 than in AGA placentas (Fig. 6B), consistent with a global metabolic shift towards coenzyme preservation.

Functionally, NAD^+^ is indispensable as a redox cofactor in glycolysis, the TCA cycle and β‐oxidation, while also serving as a substrate for sirtuins and PARPs, key regulators of oxidative stress response, DNA repair and metabolism [61, 62, 63]. The role of NAD^+^/NADH in steroidogenesis further underscores its centrality to placental adaptation. Indeed, pharmacological blockade of the salvage pathway with FK866 in PlOs reduced both ATP levels and organoid mass, highlighting the dependence of placental growth and function on the NAD^+^ salvage pathway.

Metabolomic profiling revealed reduced levels of 20β‐dehydroprogesterone, 19‐hydroxytestosterone and 16‐hydroxyestrone in SGA3, despite transcriptional upregulation of steroidogenic enzymes. This suggests that hormone output is constrained by substrate availability or post‐translational regulation. The selective reduction in 20β‐dehydroprogesterone, a weakly active progesterone metabolite, may represent a compensatory mechanism to preserve bioactive progesterone concentrations [64].

Another hallmark perturbation was the depletion of ω‐6 and ω‐3 polyunsaturated fatty acids (PUFAs), lysophospholipids (LPLs) and monoacylglycerols (MGs) in SGA3 placentas. Arachidonic acid (ARA) and docosahexaenoic acid (DHA) were significantly reduced, along with their downstream eicosanoids, including prostaglandins (PGE1, PGE2, 11‐deoxy‐PGE1) and thromboxane B_2_ (TXB_2_). The decline of both vasodilatory prostaglandins and vasoconstrictive thromboxanes highlights a global disruption of eicosanoid signalling rather than a simple shift in vascular tone, potentially impairing uteroplacental perfusion [44, 45, 65, 66]. Given DHA's established role in fetal neurogenesis, synaptogenesis and retinal development [67, 68], its depletion may have long‐term consequences for neurodevelopment in growth‐restricted offspring.

**Fig. 9:**
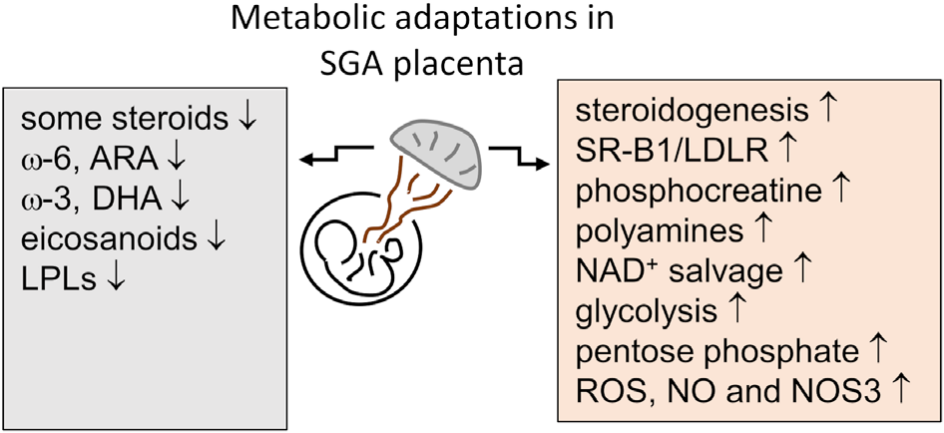
Schematic summary of altered metabolic pathways and critical metabolite levels in SGA3 placentas relative to AGA controls. Downward arrows (↓) indicate downregulation; upward arrows (↑) indicate upregulation. ARA, arachidonic acid; DHA, docosahexaenoic acid; LPLs, lysophospholipids; LDLR, low‐density lipoprotein receptor; NOS3, endothelial nitric oxide synthase (eNOS, NOS3); SR‐B1, scavenger receptor class B type 1.

Collectively, the transcriptomic and metabolomic data presented in this study, together with previous findings [16], support a model in which the SGA placenta functions as a metabolically constrained yet adaptively responsive organ. The key molecular and metabolic features characterising SGA placentas are summarised in Fig. 9. These include the following: (i) upregulation of cholesterol uptake and steroidogenic enzymes, enabling maintenance of endocrine output despite substrate limitation; (ii) activation of the NAD^+^ salvage pathway, supporting NAD‐dependent processes such as steroidogenesis, energy metabolism (particularly glycolysis) and sirtuin/PARP activity; (iii) stimulation of the pentose phosphate pathway (PPP) to preserve redox homeostasis; (iv) mobilisation of lipid reserves to offset deficits in arachidonic acid (ARA), docosahexaenoic acid (DHA) and eicosanoid biosynthesis, alterations that may impair vascular regulation and fetal neurodevelopment; (v) enhanced utilisation of arginine metabolism to generate an energy buffer in the form of creatine phosphate and increase the pool of polyamines to support growth and stress adaptation; (vi) upregulation of NOS3 expression and nitric oxide (NO) production, promoting vasodilation, improved placental perfusion and enhanced oxygen and nutrient delivery.

Current management of FGR remains largely focused on intensive antenatal surveillance and optimally timed delivery, as no therapeutic intervention has yet demonstrated the ability to reverse placental dysfunction in utero [14]. Consequently, future advances in FGR care will depend on the development of targeted strategies aimed at restoring placental function rather than solely monitoring fetal compromise. Among preventive interventions, low‐dose aspirin initiated before 16 weeks of gestation, particularly at doses ≥100 mg, has the strongest evidence for reducing the risk of placental‐mediated FGR, with a well‐established gestational age and dose‐dependent effect [69]. In contrast, low‐molecular‐weight heparin has not shown consistent benefit for FGR prevention and is therefore not recommended outside research settings [70]. To date, several investigational therapies have been explored, including phosphodiesterase type 5 inhibitors, statins, nitric oxide donors, melatonin, metformin and placental gene therapies targeting vascular endothelial growth factor or insulin‐like growth factor pathways [70]. Although preclinical and early phase studies suggest potential improvements in placental perfusion, angiogenic balance and oxidative stress, randomised clinical trials have largely failed to demonstrate clinically meaningful improvements in fetal growth or neonatal outcomes, with some interventions raising important safety concerns. In this context, the findings of this study provide a novel perspective on preventive strategies against fetal growth restriction, highlighting two promising avenues for intervention: (i) supplementation with nicotinamide adenine dinucleotide (NAD^+^) precursors, such as nicotinamide riboside (NR) or nicotinamide mononucleotide (NMN), to expand the intracellular coenzyme pool, enhance sirtuin and PARP activity and improve placental resilience [71, 72, 73, 74]; and (ii) supplementation with long‐chain polyunsaturated fatty acids (PUFAs), particularly docosahexaenoic acid (DHA), to restore eicosanoid balance, optimise uteroplacental blood flow and support fetal neurodevelopment [75, 76]. European and Asian guidelines recommend dietary intake of LC‐PUFA, particularly DHA, in women of childbearing age or during pregnancy to reduce the risk of preterm and early preterm birth [77]. Future studies will be needed to confirm whether LC‐PUFA intake may also reduce the risk of FGR.

## Materials and methods

### Sample collection

Samples were collected at the Department of Obstetrics and Gynecology, University Hospital of Udine, from May 2021 to August 2022. The study received approval from the regional ethics committee and the hospital's clinical research centre (ASUFC, decree N 289, 17/03/2021) and adhered to both the Italian Data Protection Authority's guidelines for scientific research and the ethical principles of the Declaration of Helsinki. All participating women provided written informed consent before enrolment.

All included pregnancies were monitored according to standard obstetric care and were not affected by clinically diagnosed maternal disease. Routine maternal laboratory assessments (including glucose metabolism, thyroid function, liver enzymes and blood pressure monitoring) were within pregnancy‐adjusted reference ranges. Pregnancies with any maternal condition known to directly influence placental metabolism or fetal growth, including pre‐existing or gestational diabetes, hypertensive disorders, thyroid dysfunction, cholestasis or autoimmune disease, were excluded.

Fetal growth was monitored through a series of ultrasound examinations: first‐trimester screening for chromosomal and major structural anomalies, second‐trimester anatomical assessment and two growth scans at 30 and 35 weeks' gestation. After delivery, placentas underwent both macroscopic and microscopic evaluation to confirm their suitability for the study. Inclusion criteria were maternal age ≥ 18 years, singleton pregnancy, and a live fetus at the time of ultrasound and delivery. Exclusion criteria included prenatally diagnosed fetal anomalies, maternal endocrine disorders (such as pre‐existing diabetes or thyroid dysfunction), hypertension, pregnancy complications unrelated to isolated fetal growth disorders (e.g. gestational diabetes, cholestasis), multiple pregnancies and severe maternal psychiatric conditions.

For the transcriptomic analysis, 23 term placentas were collected and categorised into three groups: appropriate‐for‐gestational‐age (AGA, *n* = 9), small‐for‐gestational age (SGA10, birth weight <10th percentile, *n* = 9), and SGA3, birth weight <3rd percentile, *n* = 5, diagnosed by ultrasound per ISUOG guidelines. Pregnancies with fetal growth restriction unrelated to placental insufficiency were excluded following routine first‐trimester screening and, when indicated, genetic testing. Placental biopsies (~1 cm^3^) were taken from the maternal side, with villous tissue isolated, thoroughly washed and immediately frozen at −80 °C. RNA‐seq sample size was determined using RNASeqPS [16]. Detailed maternal characteristics are provided in Table S1. Additionally, 3D organoids were generated from the villous tissue of three AGA and three FGR placentas, while 50% of the remaining biopsies were used for metabolomic studies.

### Placental organoid culture

Trophoblast organoids from AGA and SGA3 placentas obtained by late gestations (>32 weeks gestation) were established starting from villous tissue biopsies following the protocol previously described [16, 26]. Placental organoids (PlOs) were maintained at 37 °C, 5% CO_2_ and 21% O_2_ in TOM medium (Advanced DMEM/F12, 1× N2 supplement, 1× B27 supplement, Primocin 100 μg·mL^−1^, N‐Acetyl‐L‐cysteine 1.25 mm, L‐glutamine 2 mm, recombinant human EGF 50 ng·mL^−1^, CHIR99021 1.5 μm, recombinant human R‐spondin‐1 80 ng·mL^−1^, recombinant human HGF 50 ng·mL^−1^, A83‐01500 nm, prostaglandin E2 2.5 μm, Y‐27632 2 μm).

### Immunofluorescence

Confocal images of placental organoids (PlOs) were obtained following the protocol previously described (16). Briefly, placental organoids were fixed with 3% paraformaldehyde and permeabilised with 0.3% Triton X‐100. Actin was labelled with phalloidin‐AF546 (Molecular Probes, Eugene, OR, USA). PlOs were imaged with a confocal microscope Leica TCS SP8X. Nuclei were stained with Hoechst 33342 (10 μg·mL^−1^, Merck). Images represent maximum intensity projections of 3D image stacks and were adjusted for brightness and contrast for optimal visualisation.

### Metabolite extraction and LC–MS analysis

These experiments were carried out as previously described (16). Villous tissue samples from AGA and SGA3 placentas were analysed by liquid chromatography–mass spectrometry (LC–MS) using an ExionLC 2.0 UPLC system (SCIEX, Redwood City, CA, USA) coupled to a TripleTOF 6600+ quadrupole time‐of‐flight mass spectrometer (AB SCIEX, Redwood City, CA, USA). LIT and triple quadrupole (QQQ) scans were performed using a QTRAP LC–MS/MS system (SCIEX, MA, USA) equipped with an ESI Turbo Ion Spray interface, operating in both positive and negative ion modes and managed by Analyst 1.6.3 software (SCIEX, Ontario, Canada; RRID: SCR_015785). The ESI source parameters were: temperature 500 °C; ion spray voltage 5500 V (positive) and −4500 V (negative); ion source gases I and II at 50 psi, curtain gas at 25 psi, and high collision gas. Instrument tuning and mass calibration were carried out using polypropylene glycol solutions (10 and 100 μmol·L^−1^) in QQQ and LIT modes, respectively. Metabolite quantification was achieved via triple quadrupole mass spectrometry using multiple reaction monitoring (MRM). LC/MS analyses were conducted at Metware Biotechnology (Woburn, MA, USA). Mass spectral data were processed with Analyst 1.6.3, and quantitative analysis was performed using metwarebio software from RAW files. Missing values were imputed as one‐fifth of the minimum value for each metabolite, and only metabolites with a QC sample coefficient of variation (CV) <0.3 were retained. Principal component analysis (PCA) was performed for each pair of sample groups to assess intergroup variation. Differentially abundant metabolites between AGA and FRG groups were identified based on a variable importance in projection (VIP) score >1 (from OPLS‐DA modelling) and a Wilcoxon rank‐sum *P*‐value <0.05.

### RNA extraction, qRT‐PCR, library preparation and RNA‐sequencing

As previously described (16), placental biopsies and trophoblast organoids were lysed in TRIzol reagent (Invitrogen, Carlsbad, CA, USA). One microgram of total RNA was treated with DNase I (Ambion, USA) and reverse‐transcribed using 100 U of M‐MLV reverse transcriptase (Life Technologies, Carlsbad, CA, USA) with 1.6 μm oligo(dT) and 4 μm random hexamers (Euroclone, Milan, Italy). Quantitative RT‐PCR was performed with SYBR Green chemistry (KAPA Biosystems, Wilmington, MA, USA), and gene expression was quantified using the comparative threshold cycle method (ΔΔCt), normalising to HPRT and GAPDH. Primer sequences are provided in Table S2.

For RNA‐seq, 50 ng of total RNA was treated with DNase I, depleted of ribosomal RNA using the Ribo‐Zero method, and reverse‐transcribed into cDNA. After end repair and A‐tailing, adapters were ligated according to the DNBSEQ protocol (MGI‐Tech, Shenzhen, China). The cDNA fragments were enriched by 16 cycles of PCR amplification and purified with Ampure XP beads (Agencourt Bioscience, Beverly, MA, USA). Single‐stranded circular DNA (ssCirDNA) was generated by denaturation and circularisation, and DNA nanospheres were prepared following BGI protocols. Libraries were sequenced as 100 bp paired‐end reads at BGI Genomics (Shenzhen, China).

### RNA‐seq analysis and gene set enrichment analysis (GSEA)

Raw sequencing reads were assessed for quality using fastqc (v0.11.9) and multiqc (v1.09). Reads were aligned to the human reference genome (GRCh38, Ensembl v107) with star (v2.7.3a). Transcript assembly and quantification were performed with stringtie (v2.1.5), and read counts were extracted using the prepDE.py script. Differential expression analysis was conducted on raw gene counts with DESeq2 (R/Bioconductor). Principal component analysis, DE analysis and GSEA were performed as previously described [16].

### Cholesterol uptake assay in placental organoids using NBD cholesterol

Placental organoids were cultured in 24‐well plates and washed twice with prewarmed PBS. For the uptake assay, organoids were incubated with TOM culture medium containing 2 μm NBD cholesterol (Thermo Fisher Scientific, Waltham, MA, USA) for 20 h at 37 °C in a humidified atmosphere with 5% CO_2_. Following incubation, organoids were washed three times with cold PBS to remove excess probe. Fluorescence quantification was performed using a BioTek plate reader (Agilent Technologies, Santa Clara, CA, USA) with excitation at 485 nm and emission at 535 nm. Mean fluorescence intensity was used as a measure of cholesterol uptake. All experiments were conducted in triplicate.

### FK866 sensitivity assay in placental organoids

Placental organoids were cultured in 24‐well plates and washed twice with prewarmed PBS. For the sensitivity assay, organoids were incubated with TOM culture medium containing increasing concentrations of FK866 (Selleck Chemicals, Houston, TX, USA) for 48 h at 37 °C in a humidified atmosphere with 5% CO_2_. Cell viability was assessed using the CellTiter‐Glo Luminescent Cell Viability Assay (Promega, Madison, WI, USA) according to the manufacturer's instructions. Luminescence was measured with a BioTek plate reader (Agilent Technologies, Santa Clara, CA, USA). For PlOs mass quantification, organoids were lysed in RIPA buffer (Thermo Fisher Scientific, Waltham, MA, USA), and total protein content was quantified using the Bradford assay (Bio‐Rad Laboratories, Hercules, CA, USA). All experiments were performed in triplicate, and results were expressed as a percentage of control (untreated) organoids.

### Statistical analysis

Comparisons between two groups were performed using either Student's *t*‐test or the Mann–Whitney U test for non‐normally distributed data. For comparisons involving more than two groups, one‐way ANOVA or Kruskal–Wallis tests were applied, followed by Dunn's *post hoc* test where appropriate. Routine data analyses were conducted using Microsoft Excel (Microsoft Corp., Redmond, WA, USA) and GraphPad Prism (GraphPad Software, San Diego, CA, USA). Large‐scale data analyses were performed using R and Bioconductor packages (R Foundation for Statistical Computing, Vienna, Austria). Statistical significance was defined as *P* < 0.05 (with thresholds indicated as *P* < 0.05, *P* < 0.01, *P* < 0.001). Data are presented as mean ± standard deviation (SD) from at least three independent experiments, unless otherwise specified.

## Conflict of interest

The authors declare no conflict of interest.

## Author contributions

SX, VT and EDG performed the experiments and analysed the data; SX and LD contributed to sampling; LEX and LD supervised the project; LEX, SX and EDG planned and wrote the manuscript; EDG raised the funds.

## Supporting information


**Table S1.** Baseline characteristics of patients with appropriate‐for‐gestational‐age (AGA) and small‐for‐gestational‐age (SGA10 and SGA3) foetuses whose placental biopsies was used for the study.
**Table S2.** DNA primers used in the study.

## Data Availability

The RNA‐seq data in this study have been deposited in the GEO database with the accession code GSE262116, which is publicly available. Metabolomics raw and processed data have been deposited in Metabolights with the accession code: MTBLS10236.
