# Supplementary material for: Steroidogenic compensation and lipid deficiency with enhanced NAD + salvage in small‐for‐gestational‐age placenta
Source: FEBS J. 2026 Mar 10;293(15):4554–77. doi: 10.1111/febs.70475 (PMC13440580; doi:10.1111/febs.70475)
Supplement: Supplementary file 1 [file FEBS-293-4554-s001.pdf]

Supporting Information

Steroidogenic compensation and lipid deficiency with enhanced NAD^+ salvage in small-for-gestational-age placenta

Serena Xodo [1], Lorenza Driul [1,2], Vanessa Tolotto [2], Eros Di Giorgio [2,*] and Luigi E. Xodo [2,*]

[1] Clinic of Obstetrics and Gynecology, Santa Maria della Misericordia Hospital, ASUFC, 33100 Udine, Italy;

[2] Department of Medicine, Laboratory of Biochemistry, University of Udine, 33100 Udine, Italy;

Table S1: Baseline characteristics of patients with appropriate- for-gestational-age (AGA) and small-for-gestational-age (SGA10 and SGA3) fetuses whose placental biopsies were used for the study

N° PT	Age	GA at delivery (weeks)	IOL	Delivery mode	Fetal growth	Neonatal weight (gr)	Neonatal gender	Apgar 5'	Analysis
1	31	40+0	yes	SVD	SGA3	2720	F	9	RNA-seq, qPCR
2	31	40+1	no	SVD	SGA10	3000	F	10	RNA-seq, qPCR
3	24	41+3	yes	OVD	SGA3	2920	F	9	RNA-seq, qPCR
4	34	37+5	yes	SVD	SGA3	2350	M	9	RNA-seq, qPCR
5	32	37+1	yes	SVD	SGA3	2300	M	9	RNA-seq, qPCR
6	33	40+4	no	SVD	SGA10	3075	M	9	RNA-seq, qPCR
7	30	40+2	no	SVD	SGA10	3025	M	10	RNA-seq, qPCR
8	34	41+3	yes	OVD	SGA3	2870	M	8	RNA-seq, qPCR
10	31	38+2	yes	SVD	AGA	3205	M	9	RNA-seq, qPCR
11	36	39+1	no	SVD	AGA	3122	M	8	RNA-seq, qPCR
12	24	41+3	yes	SVD	AGA	3905	F	9	RNA-seq, qPCR
13	27	38+2	no	SVD	SGA10	2720	M	10	RNA-seq, qPCR
14	30	41+1	no	SVD	SGA10	3135	F	10	RNA-seq, qPCR
15	36	39+4	no	CD	SGA10	2960	F	9	RNA-seq, qPCR
16	34	38+3	no	SVD	SGA10	2995	F	9	RNA-seq, qPCR
17	33	38+1	no	SVD	AGA	3268	F	10	RNA-seq, qPCR
18	20	38+5	no	SVD	AGA	3170	F	10	RNA-seq, qPCR
19	29	40+2	no	SVD	SGA10	2952	F	10	RNA-seq, qPCR
20	34	38+2	no	OVD	AGA	3206	F	10	RNA-seq, qPCR
21	37	40+0	yes	SVD	AGA	3385	F	10	RNA-seq, qPCR
22	31	39+4	no	CD	AGA	3495	M	10	RNA-seq, qPCR
24	30	40+0	no	CD	SGA10	3070	M	9	RNA-seq, qPCR
33	36	39+1	no	SVD	AGA	3165	M	9	RNA-seq, qPCR
41	32	38+6	yes	SVD	SGA3	2350	M	10	Metabolomics, lipidomics, PIOs
42	40	35+3	no	SVD	SGA3	2378	F	10	Metabolomics, lipidomics, PIOs
43	22	37+5	yes	SVD	SGA3	2416	M	9	Metabolomics, lipidomics, PIOs
44	22	38+4	no	SVD	AGA	3956	M	9	Metabolomics, lipidomics, PIOs
45	33	36+4	no	CD	AGA	2860	F	10	Metabolomics, lipidomics, PIOs
46	41	38+6	yes	SVD	AGA	3100	M	9	Metabolomics, lipidomics, PIOs

GA: gestational age
IOL: induction of labor
OVD: operative vaginal delivery
SVD: spontaneous vaginal delivery
CD: cesarean delivery
FGR: fetal growth restriction
AGA: appropriate for gestational age

SGA: small for gestational age
F: female
M: male

Table S2: DNA primers used for the study

Primer name	Sequence (5'→3')	Lenght	T_m
LDLR_FW	TCTGCAACATGGCTAGAGACT	21	57.9
LDLR_RV	TCCAAGCATTCGTTGGTCCC	20	59.4
ACTB_RT_FW	CATGTACGTTGCTATCCAGGC	21	59.8
ACTB_RT_RV	CTCCTTAATGTCACGCACGAT	21	57.9
ENO1_RT_FW	AAAGCTGGTGCCGTTGAGAA	20	57.3
ENO1_RT_RV	GGTTGTGGTAAACCTCTGCTC	21	59.8
SCARB1_RT_FW	ACTTCTGGCATTCCGATCAGT	21	57.9
SCARB1_RT_RV	ACGAAGCGATAGGTGGGGAT	20	59.4
SCARB2_RT_FW	AGATGGAGATTCTTTTCACCCAC	23	58.9
SCARB2_RT_RV	CAGGAACTTTATACCGAAAGGCA	23	58.9
LSS_RT_FW	ACATTGAGGATAAGTCCACCGT	22	58.4
LSS_RT_RV	TCGTACCAGGTCAGGATCGTC	21	61.8
HPRT_RT_FW	AGACTTTGCTTTCCTTGGTCAGG	23	66.5
HPRT_RT_RV	GTCTGGCTTATATCCAACACTTCG	24	64.5
GAPD_ RT_FW	CCCTTCATTGACCTCAACTACATG	24	65.4
GAPDH_RT_RV	TGGGATTTCCATTGATGACAAGC	23	68.8
CYP11A1_RT_RV	GCAGTGTCTCGGGACTTCG	19	62.4
CYP11A1_RT_FW	GGCAAAGCGGAACAGGTCA	19	62.9
CYP19A1_RT_RV	TGGAAATGCTGAACCCGATAC	21	60.1
CYP19A1_RT_FW	AATTCCCATGCAGTAGCCAGG	21	62.2
CYP17A1_RT_RV	GCTGCTTACCCTAGCTTATTTGT	23	60.4
CYP17A1_RT_FW	ACCGAATAGATGGGGCCATATTT	23	61.3
HSD17B3_RT_FW	CTGGCGAAGTGCGTGAGATT	20	62.8
HSD17B3_RT_RV	GAGTACGCTTTCCCAATTCCAT	22	60.4
STS_RT_FW	CCTACTGTTCTTTCTGTGGGAAG	23	60.2
STS_RT_RV	CGAGGTCGTCAGCCATCAC	19	62.4
CYP1A1_RT_FW	TCGGCCACGGAGTTTCTTC	19	61.9
CYP1A1_RT_RV	GGTCAGCATGTGCCCAATCA	20	62.8
HSD3B1_RT_FW	CACATGGCCCGCTCCATAC	19	61.0
HSD3B1_RT_RV	GTGCCGCCGTTTTTCAGATTC	21	59.8
HSD17B1_RT_FW	GTGCTGGTGTGTAACGCAG	19	61.3
HSD17B1_RT_RV	GTCCCTACTACATTCACGTCCA	22	60.9
STS_RT_FW	CCTACTGTTCTTTCTGTGGGAAG	23	60.2
STS_RT_RV	CGAGGTCGTCAGCCATCAC	19	62.4
